# Transcriptome Analysis Identifies Key Metabolic Changes in the Hooded Seal (*Cystophora cristata*) Brain in Response to Hypoxia and Reoxygenation

**DOI:** 10.1371/journal.pone.0169366

**Published:** 2017-01-03

**Authors:** Mariana Leivas Müller Hoff, Andrej Fabrizius, Nicole U. Czech-Damal, Lars P. Folkow, Thorsten Burmester

**Affiliations:** 1 Institute of Zoology, Biocenter Grindel, University of Hamburg, Hamburg, Germany; 2 Department of Arctic and Marine Biology, University of Tromsø – The Arctic University of Norway, Tromsø, Norway; Scuola Superiore Sant'Anna, ITALY

## Abstract

The brain of diving mammals tolerates low oxygen conditions better than the brain of most terrestrial mammals. Previously, it has been demonstrated that the neurons in brain slices of the hooded seal (*Cystophora cristata*) withstand hypoxia longer than those of mouse, and also tolerate reduced glucose supply and high lactate concentrations. This tolerance appears to be accompanied by a shift in the oxidative energy metabolism to the astrocytes in the seal while in terrestrial mammals the aerobic energy production mainly takes place in neurons. Here, we used RNA-Seq to compare the effect of hypoxia and reoxygenation *in vitro* on brain slices from the visual cortex of hooded seals. We saw no general reduction of gene expression, suggesting that the response to hypoxia and reoxygenation is an actively regulated process. The treatments caused the preferential upregulation of genes related to inflammation, as found before *e*.*g*. in stroke studies using mammalian models. Gene ontology and KEGG pathway analyses showed a downregulation of genes involved in ion transport and other neuronal processes, indicative for a neuronal shutdown in response to a shortage of O_2_ supply. These differences may be interpreted in terms of an energy saving strategy in the seal's brain. We specifically analyzed the regulation of genes involved in energy metabolism. Hypoxia and reoxygenation caused a similar response, with upregulation of genes involved in glucose metabolism and downregulation of the components of the pyruvate dehydrogenase complex. We also observed upregulation of the monocarboxylate transporter Mct4, suggesting increased lactate efflux. Together, these data indicate that the seal brain responds to the hypoxic challenge by a relative increase in the anaerobic energy metabolism.

## Introduction

Due to the energy-demanding neuronal processes, the mammalian brain consumes a large fraction of the inhaled oxygen. Its strong oxygen-dependence makes this organ particularly sensitive towards hypoxia. In humans and most other mammals, a reduction of the oxygen supply severely impairs brain function, and prolonged hypoxia usually causes irreversible damage to the brain, for example after an ischaemic stroke, but also in other neuronal diseases [1–6]. Neurons are particularly sensitive to a shortage of oxygen due to their reliance on continuous production of metabolic energy. Reduced ATP levels eventually cause depolarization of the cell and release of excitatory neurotransmitters, which enhance Ca^2+^ influx mediated through the NMDA receptor [1, 7]. The increased cellular Ca^2+^ level induces processes among others leading to programmed cell death and enhances the inflammatory response, eventually causing irreversible brain damage. When oxygen supply is restored, additional brain damage is caused by the enhanced formation of harmful reactive oxygen species (ROS) [8].

While the brain of terrestrial mammals is usually very susceptible to hypoxia and reoxygenation, the brain of diving mammals (*i*.*e*. whales and seals) survives even extended hypoxic periods without detectable damage [5, 9–12]. Although the blood flow to hypoxia-sensitive tissues like the brain is essentially maintained during the dives [13, 14], the arterial oxygen partial pressure in the blood drops dramatically [9, 13, 15, 16] to an extent that would cause loss of consciousness in humans [17]. Paradoxically, many diving mammals are active hunters that require an active brain at low O_2_ conditions. A variety of behavioral, anatomical and physiological adaptations have evolved in seals and whales that support the brain to survive repeated breath-hold dives without detectable damage [5, 10–12, 18–22].

Still, little is known about the molecular mechanisms that contribute to the adaptation of the brain to diving. The best-studied example is the hooded seal (*Cystophora cristata*), which displays a remarkable diving capacity: Their maximum dive duration approaches 1 h while the maximum depth exceeds 1,000 m [23]. *In vitro* electrophysiological studies using brain slices from hooded seals demonstrated that their neurons remained much longer active under severe hypoxia than those from mice, in that they persisted for up to 1 h while the mouse neurons died after few minutes [24, 25]. Further, *in vitro* studies showed that the neurons in the brain of the hooded seal are also more tolerant towards low glucose and high lactate levels, under normoxia as well as under hypoxia [26]. The hypoxia tolerance of the neurons of the hooded seal brain is accompanied by the unusual localization of neuroglobin, cytochrome c and lactate dehydrogenase b in the astrocytes rather than the neurons [27–29], which indicates a higher aerobic capacity of glia cells and thus a lower dependence of neurons on O_2_. A recent comparative transcriptome approach suggested a lower aerobic energy metabolism in the seal brain compared to that of the ferret (*Mustela putorius furo*) and indicated that the stress-proteins clusterin and S100B may contribute to the hypoxia tolerance of the brain of diving mammals [30].

There are, however, still gaps in our knowledge on how the energy metabolism in the brain of diving mammals copes with the imbalance between oxygen demand and supply. An up to 3°C decrease in cerebral temperature occurs in a diving hooded seal, probably facilitating hypometabolism and reduced consumption of metabolic energy [31, 32]. On the cellular level, a reduced neuronal activity, which was observed in hooded seal neocortex slices in response to hypoxia, may represent an additional functional strategy to save energy [12, 26]. Under low oxygen conditions, the hooded seal brain may additionally profit from a higher relative contribution of anaerobic glycolysis to ATP production [9, 33]. This suggestion was reinforced by the observations that the hooded seal brain has higher glycogen stores and neuronal lactate tolerance than the mouse brain [26]. However, and apparently in contradiction to this, previous studies on the expression of glycogen phosphatase b (*Pygb*) and lactate dehydrogenase a and b (*Ldha* and *Ldhb*) failed to provide support for a higher anaerobic capacity of the seal brain [26, 29].

Here, we have employed RNA-Seq to study the response of the transcriptome of slices from the visual cortex of the hooded seal to hypoxia and reoxygenation. The electrophysiological responses of the brain slices had been investigated before [24, 26]. To better understand the molecular mechanisms that underlie the astonishing hypoxia-tolerance of the seal brain [24, 26], we analyzed the pathways and gene ontology of significantly regulated genes and the differential expression of transcripts coding for enzymes (isoenzymes and subunits) of selected pathways of the energy metabolism.

## Materials and Methods

### Ethics statement

Hooded seals (N = 3; female, 1.5–2.5 years) were captured in the pack ice of the Greenland Sea with appropriate permits from Danish and Greenland Authorities, and from the National Animal Research Authority of Norway (NARA; permits no. 5399 and 7247). Animals were handled according to the EU Directive 63 (see Directive 2010/63/EU Annex IV, point 1a), using a procedure that was approved by the NARA (permits no. 5399 and 7247) and by the authorities at the University of Tromsø (permit numbers: AAB/06). To minimize the use of animals, the tissues were employed in multiple studies [26, 29, 30, 34].

### Sample preparation

Seals were euthanized under deep gas anesthesia (ventilation with 1.5–3% isoflurane [Forene, Abbott, Germany] in air) after initial sedation (intramuscular or intravenous injection of 1.5–3.0 mg zolazepam/tiletamine [Zoletil Forte Vet, Virbac S.A., France] per kg of body mass) followed by intubation. After bleeding and subsequent decapitation, the brains of seals were removed and immediately placed in cooled (4°C) artificial cerebrospinal fluid (aCSF; 128 mM NaCl, 3 mM KCl, 1.5 mM CaCl_2_, 1 mM MgCl_2_, 24 mM NaHCO_3_, 0.5 mM NaH_2_PO_4_, 20 mM sucrose, 10 mM D-glucose) saturated with 95% O2−5% CO_2_ (normoxia).

### Preparation of brain slices and hypoxia treatment

Samples of the visual cortex were fixed with a supporting agar block to the stage of a Leica vibroslicer (VT1000s or VT1200s). Slices (400 μm thick) from the neocortex were cut and transferred into oxygenated aCSF at room temperature for at least 30 min for recovery. The slices were adjusted to 34±0.5°C for at least 20 min. Hypoxia was maintained for 60 min after switching the gas supply to 95% N2−5% CO_2_. Slices that serve as "hypoxia" samples were then preserved in RNAlater, while the "reoxygenation" samples were allowed to recover for additional 20 min in normoxia, before RNAlater preservation. The treatment scheme is given in S1 Fig. Slices that were kept under normoxia in aCSF for 80 min were used as controls. All samples were kept frozen at -20°C in RNAlater until later use.

### RNA preparation and Illumina sequencing

Total RNA was isolated from two slices per individual per experimental condition using peqGOLD Trifast (PEQLAB, Erlangen, Germany) and Crystal RNA Mini Kit column (Biolab Products, Bebensee, Germany), associated with an on-column DNA digestion with RNase-Free DNase (Qiagen, Hilden, Germany). The quantity and integrity of the RNA were verified by spectrophotometry and denaturating gel electrophoresis, and the RNA samples were used for Illumina sequencing.

Further evaluation of the RNA, cDNA library generation, and sequencing were carried out by GENterprise Genomics (Mainz, Germany). Sequencing was performed on an Illumina NextSeq 500 with Mid Output chemistry and an estimated output of 50 million reads per sample. Paired-end sequencing libraries were generated with 1.5 μg RNA from each sample. cDNA libraries with inserts of ~200–800 bp were size selected, and 150 nt paired-end sequencing was performed.

The raw Illumina data are available from the NCBI SRA database under the accession numbers SRX1567547 to SRX1567555 (Bioproject PRJNA278355). Raw sequence quality control was performed via FastQC and CLC-Genomics Workbench (version 7.5.1). For quality trimming, the following parameters were applied: all sequences with more than 2 ambiguous characters were removed. Sequences with a mean quality below a phred-value of 15 were also removed from further analysis. Additionally, the first 14 nucleotides from the 5' prime end were trimmed.

### *De novo* transcriptome assembly

For *de novo* assembly of the brain transcriptome of the hooded seal, two strategies were applied. The first assembly was generated using only the 300 nt long trimmed paired-end reads (12,473,522 reads) from seal visual cortex [30]. The second assembly was generated using, in addition, all the 150 nt paired-end reads from the hypoxia treated samples (312,047,506 reads). In both strategies, a backmapping of the reads after *de novo* assembly was performed. Only contigs with a minimum length of 300 bp were accepted. The assembly and backmapping were performed with CLC-Genomics Workbench (version 7.5.1).

### Functional annotation and comparison of transcriptomes

Annotation of the contigs of the hooded seal brain transcriptome was done with the BLAST tool of the CLC-Genomics Workbench using the SwissProt (Release date: February 2015) and the human RefSeq (Release 66, July 2014) protein databases. Only BLAST hits with E < 10^−5^ were considered significant and used for further analysis. The seal contigs were preferentially annotated using the best BLAST hits derived from the walrus (*Odobenus rosmarus*) [35] and ferret (*Mustela putorius furo*) genes [36].

Gene Ontology (GO) annotation of significant differentially expressed (DE) genes was performed with the online tool PANTHER (Protein ANalysis THrough Evolutionary Relationships; http://go.pantherdb.org), Version 10.0 Released 2015-05-15 [37]. The GO-terms in the domains "molecular function", "biological process", and "protein class" were reported. Overrepresentation of GO-terms in a certain dataset was determined using the PANTHER Overrepresentation Test (release 2015.04.30) employing the PANTHER GO-Slim terms as annotation list. *Homo sapiens* was used as the background reference list for statistical calculation of overrepresentation of GO-Terms. GO-terms with a p-value ≤ 0.05 after Bonferroni correction were considered as significant.

For principal component analysis (PCA) the log2 transformed RPKM values (Reads Per Kilobase exon model per Million mapped reads) of either all expressed transcripts (9321 features with RPKM>1) or the statistically DE transcripts shared between all samples (109 features) were used. A projection scatter plot was generated using CLC Genomics Workbench (version 7.5.1).

The hierarchical clustering was calculated using the log2 transformed RPKM values for either all statistically differentially expressed transcripts shared between all samples (109 features) or only the top 40 differentially expressed transcripts (e.g. top 20 upregulated and top 20 downregulated shared between normoxia, hypoxia, and reoxygenation). For the feature clustering the "Manhattan distance" was used to calculate the average linkage. A heat map of feature clustering was calculated using CLC Genomics Workbench (version 7.5.1).

### Expression analysis (RNA-Seq)

The mapping of the reads was performed using the RNA-Seq algorithm of the CLC-Genomics Workbench (version 7.5.1). The genome of the ferret (*Mustela putorius furo*), Build 1.0.75, was used as reference. The trimmed reads were mapped using the following parameters: 75% of the read and 75% of all nucleotides were required to match the reference for the read to be included in the mapping. The paired read distance was calculated automatically. To eliminate repetitive sequence bias in the quantification only reads that uniquely map in the genome were used for the calculation of the RPKM value. Statistically significant expression changes in the differentially treated slices were calculated by an empirical analysis of digital gene expression using the CLC-Genomics Workbench. The tool implemented the "Exact Test" [38] and was used with the unique gene reads as count values with a cut-off of 5 reads. To correct for multiple testing, a Bonferroni correction of the p-values was applied. Only genes with a fold change ≥2 and a Bonferroni-corrected p-value ≤ 0.01 were considered as significant.

For the analysis of the expression of genes coding for energy metabolism enzymes (isoenzymes and enzyme subunits are listed in S3 Table) in normoxia, hypoxia, and hypoxia/reoxygenation, trimmed reads were additionally mapped to the ferret mitochondrial chromosome (RefSeqID: NC_020638.1) using the same settings as described above. The differences in RPKM values of nuclear and mitochondrially encoded genes in normoxia, hypoxia, and hypoxia/reoxygenation samples were statistically compared using One-way Analysis of Variance (ANOVA), and Tukey's or Fisher's LSD multiple comparison tests available in GraphPad Prism 6 (La Jolla, CA, USA). Note that the RPKM values of nuclear and mitochondrially encoded genes were obtained from two different mappings and thus the numbers cannot be directly compared.

### Quantitative real-time reverse transcriptase-polymerase chain reaction (qRT-PCR)

The expression levels of selected genes were verified by qRT-PCR. Total RNA purification and cDNA synthesis was carried out as previously described [29]. qRT-PCR was performed on an ABI 7500 real-time PCR system with the Power SYBR Green master mix (Applied Biosystems, Darmstadt, Germany) using the following protocol: 15 sec 95°C, 15 sec 60°C, 30 sec 72°C, 40 cycles. The mRNA copy number in a cDNA sample equivalent to 25 ng total RNA was determined using a concentration curve of the recombinant plasmid as standard, and then was normalized to 1 μg of total RNA.

## Results

### Transcriptomes from the hooded seal brain and brain slices

In a previous study [30], we generated 37,108,070 Illumina reads from the untreated visual cortex of the hooded seal (*C*. *cristata*) (Table 1). To elucidate the molecular adaptation of the seal brain to hypoxic challenge, as it occurs at least during long dives, we examined the gene expression changes in brain slices of the hooded seal after hypoxic treatment for 60 min following reoxygenation for 20 min (S1 Fig). A total of 437,291,176 new Illumina reads were obtained, reflecting three biological replicates from each, the normoxia control, hypoxia-treated slices and hypoxia-reoxygenation treated slices (Table 1).

**Table 1:** Summary of Illumina sequencing of the hooded seal brain transcriptomes.

Normoxia	SRA accession	Raw Reads	Reads after Trimming	Mapped Reads %
Ccr2N_S1	SRR3156151	48,820,474	35,722,796	50.64
Ccr3N_S2	SRR3156152	54,006,392	40,570,390	51.38
Ccr4N_S3	SRR3156153	62,553,574	48,235,762	52.24
**Hypoxia**				
Ccr2H60_S4	SRR3156154	48,028,568	33,531,186	50.11
Ccr3H60_S5	SRR3156155	44,978,692	30,974,474	47.13
Ccr4H60_S6	SRR3156156	43,793,918	31,045,780	46.32
**Reoxygenation**				
Ccr2R20_S7	SRR3156157	42,408,488	30,576,200	51.12
Ccr3R20_S8	SRR3156158	45,364,920	30,814,718	51.04
Ccr4R20_S9	SRR3156159	47,336,150	30,576,200	46.36
Ccr2-Cortex	SRR3001184	37,108,070	12,473,522	52.45

The number of reads before and after quality trimming is given. The percentage of reads mapped to the ferret genome is denoted.

Two different assemblies were generated, consisting of the trimmed reads from the untreated visual cortex only (assembly #1) or combining the trimmed reads from the visual cortex with the treated slices from the visual cortex (assembly #2). As expected, the number of contigs increased from 85,821 in assembly #1 to 149,112 in assembly #2 (Table 2). We annotated the contigs ≥500 bp from each assembly (60,602 and 89,777 contigs, respectively) using the SwissProt and the human RefSeq databases by blastx. The numbers of significant blastx hits results were only slightly higher for assembly #2 (Table 2). However, a comparison of the results performed with the human RefSeq database showed that among the 23,095 unique blastx-hits combined in both assemblies, only 12,265 were shared by both assemblies (S2 Fig). Assembly #1 produced 4,839 unique blastx hits only found there and 5,991 hits were found only in assembly #2. Thus assembly #2, which contained the larger number of contigs and more blastx hits, was used for further analysis.

**Table 2:** Summary of the *de novo* assembly of the hooded seal brain transcriptomes.

	Assembly #1 (visual cortex only)	Assembly #2 (visual cortex + slices)
Reads	12,473,522	328,963,770
Contigs	85,821	149,112
Singletons	1,919	234
>1000 nt	23,481	34,038
N50	1,470	1,194
Max	22,647	22,727
Average	1,061	908
Contigs for BLASTx (≥500 nt)	60,602	89,777
BLASTx Swissprot (E<1e-5)	23,224	24,143
blastx human RefSeq (E<1e-5)	22,134	23,039

The assemblies were obtained from the visual cortex (MiSeq), and from the visual cortex (MiSeq) + brain slices reads (NextSeq).

### Transcriptome response of seal brain slices to hypoxia and reoxygenation

**Fig 1:**
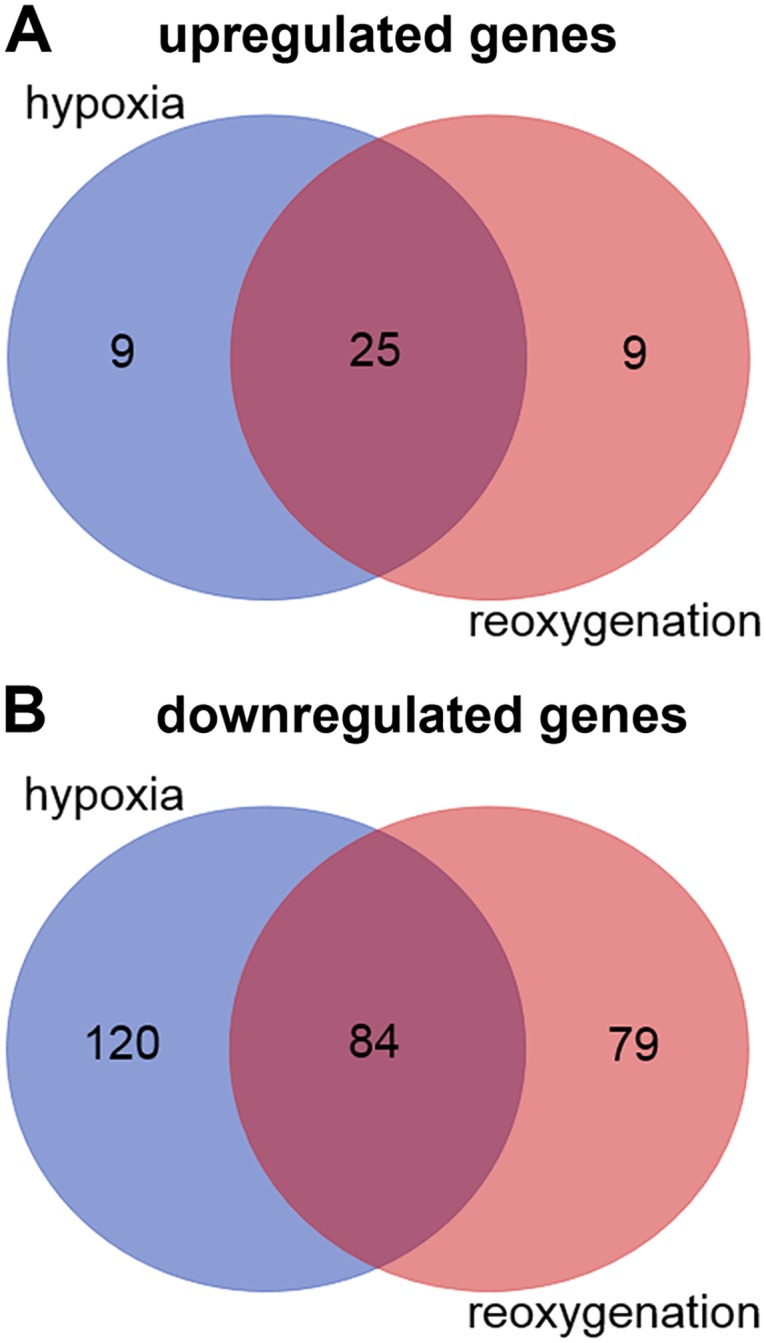
Venn diagram showing the unique and shared DE genes in hypoxia and hypoxia/reoxygenation treated brain slices of the hooded seal. (A) Upregulated genes, (B) downregulated genes.

**Fig 2:**
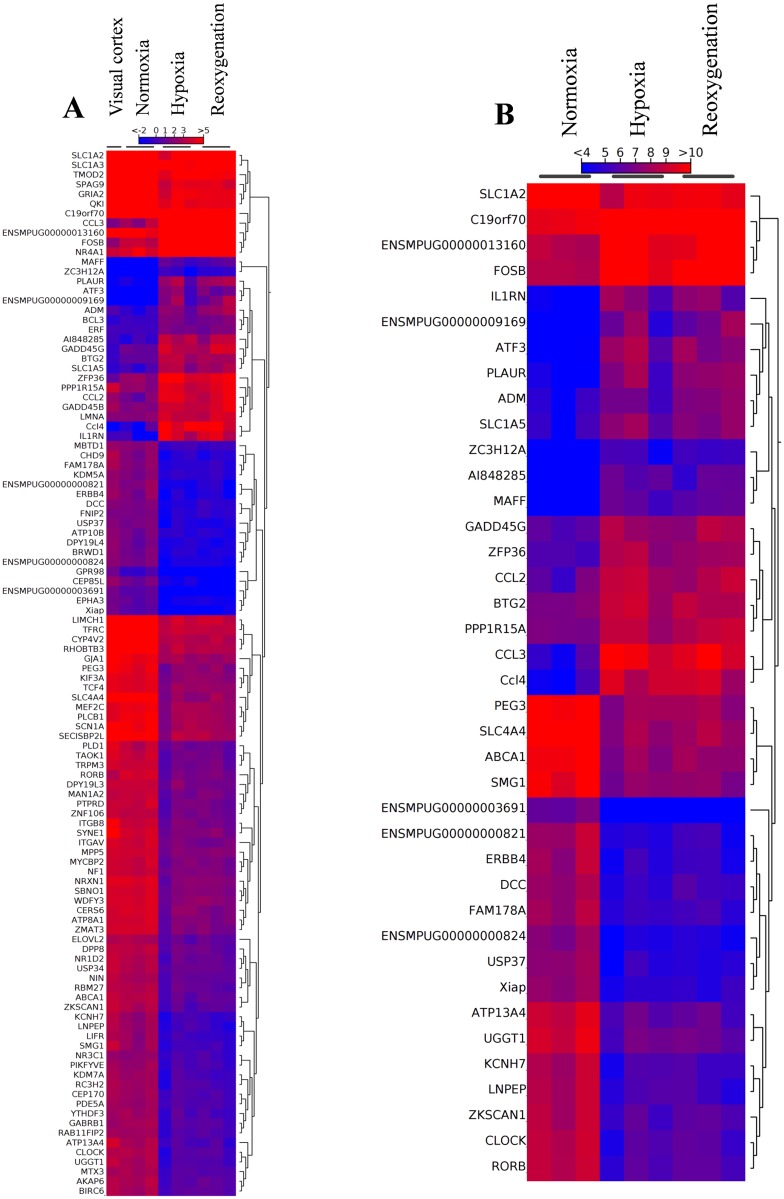
Heat map of gene expression in the visual cortex and brain slices from the hooded seal. (A) Hierarchical clustering of the regulated genes in the differentially treated brain slices and the visual cortex. (B) Hierarchical clustering of the top 20 up- and top 20 downregulated genes shared between hypoxia/reoxygenation and the normoxia control.

Hypoxia treatment of the brain slices for 60 min causes the significant upregulation of 34 genes and the downregulation of 204 genes (S1 Table). After additional 20 min reoxygenation, 34 genes were significantly upregulated and 163 genes downregulated (S2 Table). 25 of the genes were found upregulated in both the hypoxia and the hypoxia/reoxygenation slices; among the downregulated genes, 84 were shared by the hypoxia and the hypoxia/reoxygenation samples (Fig 1). There were no significantly differentially expressed genes when the transcriptomes from the hypoxia and the hypoxia/reoxygenation slices were compared. Hierarchical clustering of the differentially regulated genes (Fig 2A) showed that the gene expression levels in the normoxia control resemble that of the untreated visual cortex, demonstrating the validity of the normoxia controls used for the brain slices. The heatmap (Fig 2B) and the principal component analysis (S3 Fig) visualize the similarity of the responses in the brain slices treated for 60 min with hypoxia and the slices after 60 min hypoxia and 20 min reoxygenation.

The most strongly induced gene upon hypoxia is the cytokine interleukin-1β (*Il1b*), a mediator of the inflammatory response, which was 668.85 times higher expressed in the hypoxic brain slices (Table 3). We also observe strong upregulation (~25 to 65-fold) of other cytokines/chemokines (*Ccl3*, *Ccl4*, *Cxcl1*, and *Il1rn*). Other prominently upregulated genes include the urokinase receptor *Plaur*, which is involved in wound healing, and the transcription factors *Fosb* and *Atf3*. Of the 204 significantly downregulated genes, the zinc finger transcription factor *Znf211* showed the strongest response (113.94-fold) together with other transcription factors, like *Znf425* and *Klhl11* (Table 3).

**Table 3:** Annotation of hypoxia-regulated genes.

**A. Most strongly up-regulated**	**Gene symbol**	**Function**	**Fold change**
Interleukin-1 beta	*Il1b*	cytokine	668.85
chemokine (C-C motif) ligand 3	*Ccl3*	cytokine	65.23
chemokine (C-C motif) ligand 4	*Ccl4*	cytokine	55.42
v-maf avian musculoaponeurotic fibrosarcoma oncogene homolog F	*Maff*	transcription factor	45.82
interleukin 1 receptor antagonist	*Il1rn*	cytokine	36.92
activating transcription factor 3	*Atf3*	transcription factor	35.98
growth-regulated alpha protein-like	*Cxcl1*	cytokine	26.55
plasminogen activator, urokinase receptor	*Plaur*	receptor	18.47
Shisa family member 8	*Shisa8*	-	15.57
FBJ murine osteosarcoma viral oncogene homolog B	*Fosb*	transcription factor	12.61
**B. Most strongly down-regulated**	**Gene symbol**	**Function**	**Fold change**
zinc finger protein 211	*Znf211*	transcription factor	-113.94
stonin 2	*Ston2*	endocytosis	-28.17
zinc finger protein 425	*Znf425*	transcription factor	-11.46
solute carrier family 4 (sodium bicarbonate cotransporter), member 4	*Slc4a4*	transporter	-7.95
sacsin molecular chaperone	*Sacs*	chaperone	-7.9
dmX-like protein 1	*Dmxl1*	-	-7.74
kelch-like family member 11	*Klhl11*	transcription factor	-7.12
low density lipoprotein receptor-related protein 5-like	*Lrp5*	receptor	-6.69
v-erb-b2 avian erythroblastic leukemia viral oncogene homolog 4	*Erbb4*	receptor tyrosine kinase	-6.64
pleckstrin homology domain containing, family H member 2	*Plekhh2*	cytoskeleton	-6.32

Most strongly up- (A) and down- (B) regulated genes in hooded seal brain slices after 1 h hypoxia (N = 3). The full list of significantly regulated genes is provided in S1 Table.

After reoxygenation, *Il1b* was again the strongest responding gene (425.38-fold upregulation) (Table 4). In general, the list of the top differentially expressed genes resembles that of the response to hypoxia, including the cytokines/chemokines (*Ccl3*, *Ccl4*, *Cxcl1*, and *Il1rn*) and the transcription factors *Fosl1* and *Atf3*. The downregulated genes cover a variety of genes. The strongest response was found for the mRNA of the motor protein *Kif11* (-9.32) and the transcription factor *Zdbf2* (-8.95) (Table 4).

**Table 4:** Annotation of hypoxia/reoxygenation-regulated genes.

**A. Most strongly up-regulated**	**Gene symbol**	**Function**	**Fold change**
Interleukin-1 beta	*Il1b*	cytokine	425.38
FOS-like antigen 1	*Fosl1*	transcription factor	374.32
thyrotropin-releasing hormone	*Trh*	hormone	111.86
chemokine (C-C motif) ligand 3	*Ccl3*	cytokine	51.97
v-maf avian musculoaponeurotic fibrosarcoma oncogene homolog F	*Maff*	transcription factor	48.23
chemokine (C-C motif) ligand 4	*Ccl4*	cytokine	44.6
growth-regulated alpha protein-like	*Cxcl1*	cytokine	38.1
BCL2-related protein A1	*Bcl2a1*	signalling	35.03
interleukin 1 receptor antagonist	*Il1rn*	cytokine	30.69
activating transcription factor 3	*Atf3*	transcription factor	28.73
**B. Most strongly down-regulated**	**Gene symbol**	**Function**	**Fold change**
kinesin family member 11	*Kif11*	motor protein	-9.32
zinc finger, DBF-type containing 2	*Zdbf2*	transcription factor	-8.95
low density lipoprotein receptor-related protein 5	*Lrp5*	receptor	-8.64
centrosomal protein 85kDa-like	*Cep85l*	-	-8.16
RAR-related orphan receptor A	*Rora*	transcription factor	-7.14
formin 1	*Fmn1*	cytoskeleton	-6.95
large tumor suppressor kinase 1	*Lats1*	enzyme	-6.84
zinc finger protein 197	*Znf197*	transcription factor	-6.66
tet methylcytosine dioxygenase 2	*Tet2*	enzyme	-6.60
matrix metallopeptidase 16	*Mmp16*	metalloprotease	-5.93

Most strongly up- (A) and down- (B) regulated genes in hooded seal brain slices after 1 h hypoxia followed by 20 min reoxygenation (N = 3). The full list of significantly regulated genes is provided in S1 Table.

### Gene ontology analyses of regulated genes in seal brain slices

**Fig 3:**
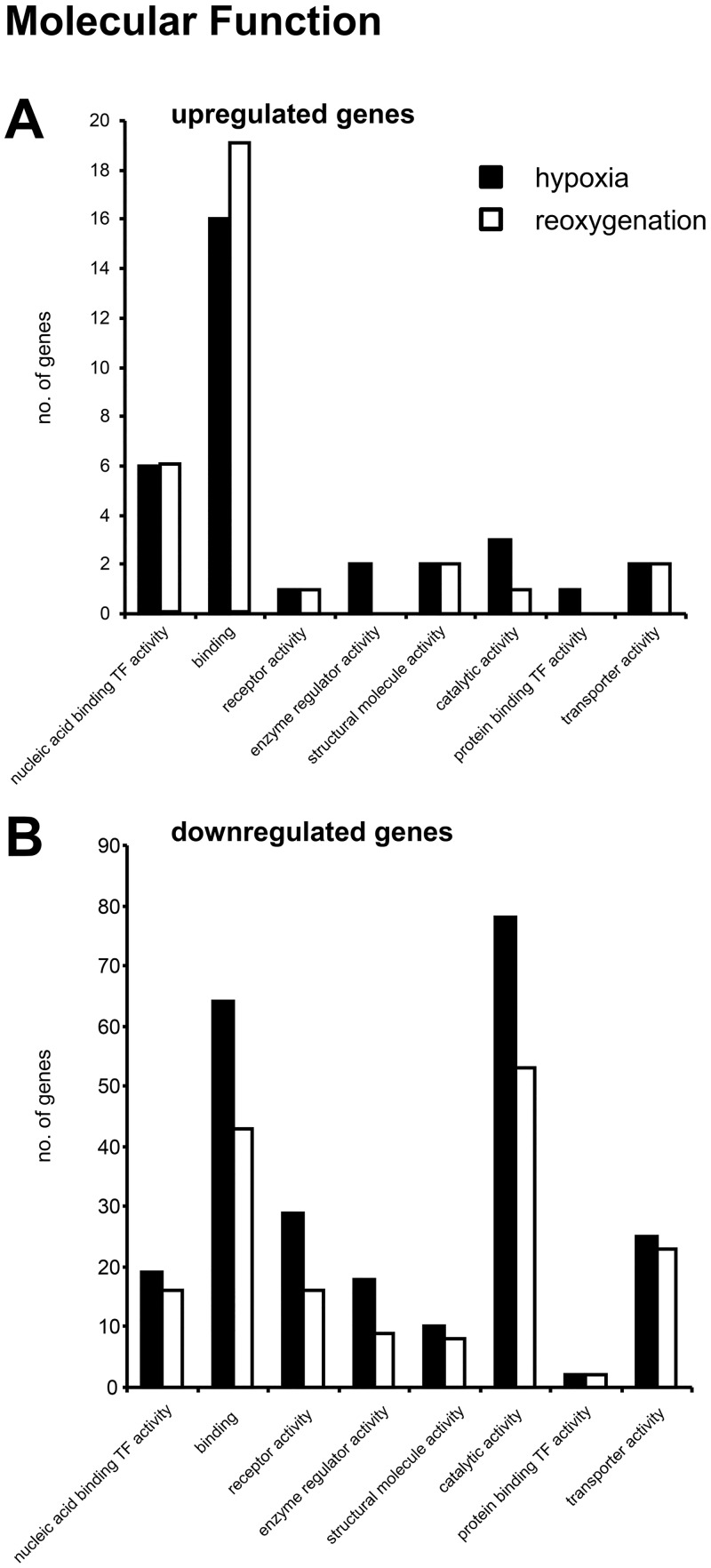
Gene ontology analyses of hypoxia and hypoxia/reoxygenation-regulated genes in seal brain slices. GO analysis in the domain "molecular function" of the significantly up- (A) and down- (B) regulated genes in the hooded seal brain slices after 1 h hypoxia (black) and after 1 h hypoxia followed by 20 min reoxygenation (white). The analyses of the GO terms in the categories "biological process" and "protein class" are given in S3 and S4 Figs.

Among the genes upregulated in the seal brain slices after hypoxia-treatment, the GO terms "binding" (GO:0005488) (16 genes), which partly reflects the large number of cytokines/chemokines, and "nucleic acid binding transcription factor activity" (GO:0001071) (6 genes) had the highest numbers in the domain "molecular function" (Fig 3A). A PANTHER Overrepresentation Test showed >5-fold, significant enrichment in the terms "chemokine activity" (GO:0008009; p = 0.00379) and "cytokine activity" (GO:0005125; p = 0.00372). In the domain "biological process", none of the terms was significantly overrepresented. In the domain "protein class", the terms "nucleic acid binding" (PC00171) (7 genes), "signaling molecule" (PC00207) (7 genes) and "transcription factor" (PC00218) (6 genes) had the highest numbers (S5 Fig). In agreement with the results in the domain "molecular function", the PANTHER Overrepresentation Test found a >5-fold enrichment of the terms "chemokine" (PC00074; p = 0.0113) and "cytokine" (PC00083; p = 0.00208). KEGG pathway analysis of the hypoxia-upregulated genes identified the MAPK (mitogen-activated protein kinases) pathway as significantly enriched.

GO analyses found the terms "binding" (64 genes) and "catalytic activity" (GO:0003824) (78 genes) to reflect the largest number of genes downregulated under hypoxia in the domain "molecular function" (Fig 3B). The PANTHER Overrepresentation Test showed significant enrichment (thus downregulation) of "phosphoprotein phosphatase activity" (GO:0004721; 4.9-fold; p = 0.0191), "ion channel activity" (GO:0005216; 3.8-fold; p = 0.016), "transporter activity" (GO:0005215; 2.42-fold; p = 0.00727), "transmembrane transporter activity" (GO:0022857; 2.32-fold; p = 0.0381) and "catalytic activity" (GO:0003824; 1.6-fold; p = 0.00052). In the domain "biological process", the GO term "cellular process" (103) and "metabolic process" (GO:0008152) (103) were the highest (S4 Fig). The PANTHER Overrepresentation Test found several GO terms to be enriched among the significantly regulated genes, including "ion transport" (GO:0006811; 2.99-fold; p = 0.00334), "nervous system development" (GO:0007399; 2.85-fold; p = 0.00252) and "cellular protein modification process" (GO:0006464; 2.59-fold; p = 0.000166). The highest number of GO terms in the domain "protein class" were "receptor" (PC00197) (31 genes), "transporter" (PC00227) (24 genes), "nucleic acid binding" (PC00171) (24 genes) and "hydrolase" (PC00121) (24 genes) (S5 Fig). The terms "ligand-gated ion channel" (PC00141; >5-fold; p = 0.0178), "transporter" (PC00227; 2.51-fold; p = 0.00712) and "receptor" (2.07-fold; p = 0.0184) were found significantly enriched in a PANTHER Overrepresentation Test. KEGG analysis of the downregulated genes identified mostly pathways related to neuronal processes: "dopaminergic synapse", "axon guidance", "retrograde endocannabinoid signaling", "glutamatergic synapse" and "arrhythmogenic right ventricular cardiomyopathy".

A similar pattern of GO terms was found in genes differentially expressed after hypoxia/reoxygenation. A significant enrichment in the domain "molecular function" (Fig 3A) was found for the terms "chemokine activity" (>5-fold; p = 0.00379), "cytokine activity" (>5-fold; p = 0.00372) and "binding" (2.3-fold; p = 0.00767). In the domain "biological process" (S4 Fig), the terms "response to stimulus" (GO:0050896; 3.52-fold; p = 0.0287) and "cellular process" (2.07-fold; p = 0.0254) were significantly overrepresented. In the domain "protein class" (S5 Fig), a >5-fold enrichment of the terms "chemokine" (p = 0.0103), "cytokine" (p = 0.00176) and "signaling molecule" (p = 0.00298) was found by the PANTHER Overrepresentation Test. KEGG analysis identified the pathways "rheumatoid arthritis", "NF-kappa B signaling pathway", "malaria" and "TNF signaling pathway" as significantly affected by hypoxia/reoxygenation.

GO-analyses of the genes downregulated by hypoxia/reoxygenation (Fig 3B) found in the domain "molecular function" a significant enrichment of the terms "ion channel activity" (4.01-fold; p = 0.0388), "transporter activity" (2.82-fold; p = 0.00122) and "transmembrane transporter activity" (2.81-fold; p = 0.00335). In the domain "biological process" (S4 Fig), several GO terms were found significantly regulated; among those were "ion transport" (3.41-fold; p = 0.00146), "cellular process" (3.41-fold; p = 0.000005) and "primary metabolic process" (GO:0044238; 1.47-fold; p = 0.0134). In the domain "protein class" (S5 Fig), the terms "transporter" (2.99-fold; p = 0.00058), "cation transporter" (>5-fold; p = 0.00112) and "ion channel" (4.13-fold; p = 0.0368). No significant enrichment among the reoxygenation-downregulated genes was found in the KEGG-pathway analysis.

### Gene expression changes in the energy metabolism

We specifically analyzed the expression changes of 165 selected genes coding for enzymes and other proteins that are involved in the cellular energy metabolism (S3 Table; S6 Fig). For 22 genes, no annotations were available in the current version of the ferret genome [36], and one was not found in our dataset; these 23 genes were not considered. We found in the hooded seal brain transcriptome 142 genes that encode enzymes related to the cellular energy production. For the evaluation of gene expression, the ferret nuclear and mitochondrial genomes were used as a guideline. We removed 18 nuclear-encoded genes with <1 RPKM and one mitochondrial gene (*MtNd4l*) with very low expression value from the dataset, leaving 123 genes for further analyses.

**Fig 4:**
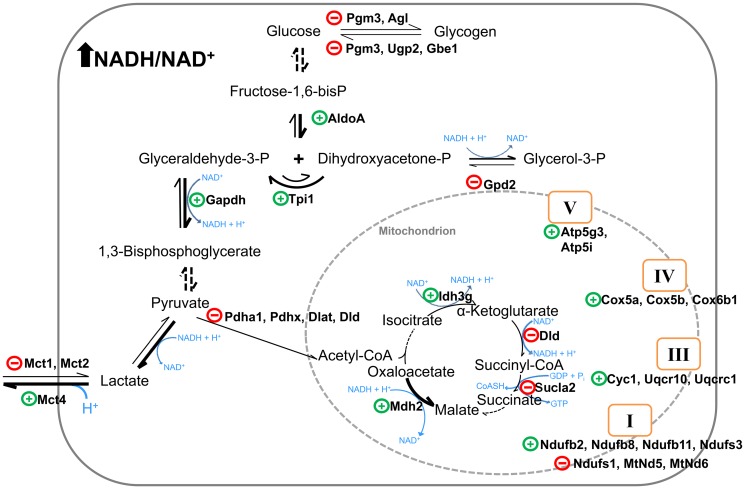
Regulation of enzymes of the energy metabolism enzymes in hooded seal cortex in response to hypoxia and reoxygenation. Upregulated genes are indicated by a green plus (+), downregulated genes are indicated by a red minus (-). All genes show the same patterns of regulation, except *Cox5a* and *Cox5b*, which were only regulated by hypoxia, and *Atp5i*, *Sucla2*, and *Idh3g*, which were only found regulated after reoxygenation. Thick arrows represent the reactions that may be favored by the regulations observed here. See S3 Table for abbreviations and details.

**Fig 5:**
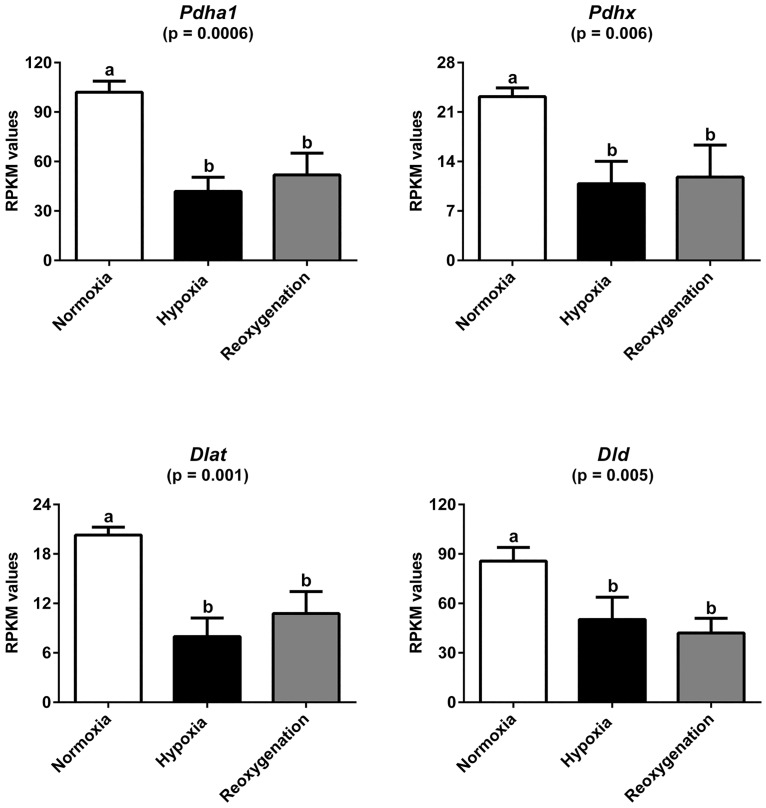
Expression levels of components of the pyruvate dehydrogenase (PDH) complex. The RPKM values of *Pdha1*, *Pdhx*, *Dlat* and *Dld* show significant downregulation in the seal brain slices after hypoxia and hypoxia/reoxygenation (N = 3). Error bars represent standard deviations. Statistical significance was indicated by one-way ANOVA associated with Tukey's multiple comparison tests; different letters indicate statistically significant differences.

We found significant upregulation upon hypoxia and hypoxia/reoxygenation of 18 genes and downregulation of 15 genes that are involved in the energy metabolism (Fig 4; S3 Table). Among those, we found upregulation of three genes involved in glucose metabolism (aldolase A [*AldoA*], triose phosphate isomerase [*Tpi1*], and glyceraldehyde-3-phosphate dehydrogenase [*Gapdh*]), and downregulation of four of the five pyruvate dehydrogenase complex subunits (pyruvate dehydrogenase (lipoamide) alpha 1 [*Pdha1*], E3 binding protein [*Pdhx*], dihydrolipoamide S-acetyltransferase [*Dlat*] and dihydrolipoamide dehydrogenase [*Dld*]) (Fig 5). Further, we analyzed the mRNA levels of two selected genes, *Pdha1* and *Dlat* by qRT-PCR, demonstrating an excellent correlation with the data from RNAseq (S7 Fig).

The analyzed genes coding for the enzymes of the tricarboxylic acid cycle and the components of the mitochondrial electron transport chain showed no unique pattern, in terms of up- and downregulation of pathways. The monocarboxylate transporter Mct4 (*Slc16a3*), which facilitates the export of lactate, was found upregulated while the monocarboxylate transporters Mct1 (*Slc16a1*; *ENSMPUG00000005243*) and Mct2 (*Slc16a7*) were downregulated upon hypoxia and reoxygenation. Unexpectedly, we observed a decrease in the RPKM values of the glycogen metabolism enzymes: UDP-glucose pyrophosphorylase (*Ugp*), glycogen branching enzyme (*Gbe1*), phosphoglucomutase 3 (*Pgm3*), and glycogen debrancher enzyme (*Agl*) upon hypoxia and hypoxia/reoxygenation. Also noteworthy is the lack of significant differences in the expression of typical glycolysis rate-limiting enzymes, such as hexokinase-1 (*Hk1*), phosphofructokinase-1 (*Pfkm*, *Pfkl*, and *Pfkb*), and lactate dehydrogenase (*Ldha* and *Ldhb*) between normoxia, hypoxia and hypoxia/reoxygenation (S3 Table).

## Discussion

### Hypoxia-response of the brain of diving mammals

In most humans and other terrestrial mammals, cerebral hypoxia is usually a non-physiological or pathological situation that brings about a broad range of physiological and molecular changes that may help the brain to survive [1, 3, 39]. By contrast, the brain of diving mammals has to deal regularly with O_2_ conditions that would compromise normal brain function in terrestrial mammals [5, 9–12]. Notably, the brains of seals and whales not only survive these conditions without obvious damage but remain functional. This observation has sparked a number of studies on the remarkable features of the diving brain, which sets it aside from the brains of most non-diving mammals [for review, see 5, 10, 11, 12, 18, 20].

We used brain slices from the hooded seal (*C*. *cristata*) to investigate the response to hypoxia and reoxygenation at the molecular level. The brain-slice technique had been applied before to compare the performance of the hooded seal neurons with those of a mouse under hypoxia and reoxygenation using electrophysiological methods *in vitro* [24–26]. Although the electrophysiological studies showed differences between the hypoxia and reoxygenation states [26], we observed little changes in the gene expression patterns of seal slices after 60 min hypoxia compared with slices after 60 min hypoxia followed by 20 min reoxygenation (Figs 2 and 3). After correction for multiple testing, none of the observed differences were significant. This finding indicates that–within the time frame of our exposure protocols–there was no detectable specific reoxygenation response at the gene expression level. Obviously, 20 min reoxygenation was not sufficient for the brain slices to return to the normoxic state of gene expression, suggesting that posttranscriptional processes play an important role in modulating electrophysiological activity [24–26]. In the following, the results from the hypoxia and reoxygenation responses are discussed together.

### The response of the seal brain to hypoxia and reoxygenation is an active and specific process

In summary, hypoxia and hypoxia/reoxygenation increased the expression of 18 unique and 25 shared genes and reduced the expression of 283 genes in the hooded seal brain slices; 84 of them were shared between the treatments (Fig 1). Nevertheless, the total mRNA expression levels (as measured by the sum of the RPKM values or the number of sequenced reads for each sample) were not significantly different in the control and treatment samples, indicating that the hypoxia and hypoxia/reoxygenation treatments applied here do not cause a general repression of gene expression. The differential induction and repression of genes further indicate that the response of the hooded seal brain to the hypoxic challenge is an active process rather than a passive reduction of cellular metabolism. Probably, none of the regulated genes alone is responsible for the observed tolerance of brain slices of the hooded seal towards hypoxia and hypoxia-related stresses [24–26]. Rather, the specific combination of intrinsically high levels of stress genes, such as clusterin and S100B [30], along with the regulation of specific metabolic and regulatory pathways, is probably required for the overall hypoxia tolerance.

### Hypoxia and reoxygenation induce a conserved mammalian stress response in the hooded seal brain

The most prominent group of genes that were upregulated in response to the hypoxia and hypoxia/reoxygenation treatments include cytokines and immediate early genes, for example, *Fosb*, which is a component of the AP-1 transcription factor (Tables 3 and 4; S1 and S2 Tables). Inflammation processes are known to commence within hours after an ischemic insult [40, 41] and are involved in the removal of apoptotic and necrotic cells after ischemia [42]. This may be interpreted as an indication that neural damage such as cell death occurs under the applied experimental conditions, although it is unknown (but unlikely) whether such extreme conditions also occur in the intact seal brain during diving.

The general pattern in the brain slices of the hooded seal resembles the conserved response of the mammalian brain to stroke [39]. In each of the two treatment groups, 14 genes (out of 43) that were found upregulated were also found previously to be induced in the rat brain after 15 min global ischemia, followed by reperfusion [39]. These mainly include the cytokines/chemokines and transcription factors, which are probably components of a conserved stress response mechanism of the mammalian brain. However, among the downregulated genes only six (after hypoxia) and three genes (after hypoxia/reoxygenation), respectively, were overlapping with the previous rat study, emphasizing the differences between the treatments and divergent strategies.

### Switches in the energy metabolism of the seal brain in response to hypoxia

The energy metabolism is an essential process, and its specific regulation probably helps the seal brain to survive better the challenges of the reduced availability of O_2_. Under hypoxic conditions, glycogen stores in the hooded seal brain may support glycolysis, in addition to the glucose supplied by the maintained cerebral blood supply during diving [18, 20]. However, our data failed to indicate any activation of glycogen metabolism (Fig 4). This may be due to the presence of 10 mM glucose in the slices artificial medium, which could have had a repressive effect on the genes of the glycogen metabolism. This may mimic the situation of the intact animal, in which glucose supply to the brain is maintained by the blood flow. However, further experimental data are required to support this interpretation.

We also did not observe notable changes in the expression of genes that code for the enzymes that are generally considered as rate-limiting in glycolysis [43]: hexokinase (*Hk1*), phosphofructokinase-1 (*Pfkm*, *Pfkl*, *Pfkb*) pyruvate kinase (*Pkm*) and lactate dehydrogenase (*Ldha* and *Ldhb*) (S3 Table). However, we found that the levels of three other glycolytic genes, *AldoA*, *Tpi1* and *Gapdh*, increased in the slices challenged with hypoxia and hypoxia/reoxygenation (Fig 4), which may, in fact, indicate an enhanced glycolytic rate.

An increased reliance on glycolytic energy production during low oxygen conditions is also indicated by the downregulation of the monocarboxylate transporter *Mct2* and the upregulation of *Mct4*. Mct2 has a high affinity to lactate and pyruvate and is expressed mainly in tissues that use lactate as a substrate for aerobic metabolism or gluconeogenesis [44]. Mct4 is mainly present in tissues that rely on anaerobic glycolysis. It has a low affinity to pyruvate, which is crucial to regenerate NAD^+^ for glycolysis by ensuring the availability of pyruvate. Pyruvate is reduced to lactate, which is then transported out of the cell by Mct4. Previous studies suggested that Mcts are involved in the response to hypoxia and ischemia. For example, hypoxic preconditioning caused an upregulation of Mct4 in rat astrocytes [45] and a knockdown of *Mct4* interfered with the survival of mixed astrocyte-neuron cultures under hypoxia [46]. Mct4 activity and structure appear to be associated to the astrocyte-specific excitatory amino acid transporter 1 (Eaat1) [47].Therefore, the regulations of the Mcts observed in the seal brain slices may indicate an adaptation to the efflux of lactate produced by the anaerobic glycolysis. In addition, the increased expression of *Mct4* may be part of the molecular mechanisms of the tolerance towards hypoxia and lactate in hooded seal brain slices [26]. In summary, the data indicate a stimulation of the glycolysis pathway by substrate flux. Previous hypotheses on an altered labor division between astrocytes and neurons in diving mammals [27, 28] would imply differential cellular responses also in these terms. However, the approach did not allow us to discriminate between astrocytes and neurons.

Hypoxia and reoxygenation also affect the genes coding for mitochondrial proteins. Compared to normoxia, the mRNA levels of the subunits of the pyruvate dehydrogenase complex (Pdc), *Pdha1*, *Dlat*, *Dld* and *Pdhx*, notably decreased in the seal brain slices under hypoxia by 60%, 55%, 37% and 41%, respectively (Fig 5). After reoxygenation, *Pdha1*, *Dlat*, *Dld* and *Pdhx* levels remained low (Fig 5). The Pdc is the gatekeeper for glucose oxidation; therefore, the downregulation of Pdc subunits suggests that oxidation in the mitochondria is repressed. Other studies already showed that Pdc inhibition is a fundamental adaptive mechanism of the mammalian energy metabolism to hypoxia [48, 49]. In cultured human and mice cancer-cells, the decreased pyruvate dehydrogenase activity during hypoxia has been related to a reduced ROS production and to cell death resistance [48]. For the enzymes that are involved in the tricarboxylic acid cycle and the components of the respiratory chain constituents, no clear pattern was observed. Reductions in activity and the content of mitochondrial proteins of different mammalian cells exposed to hypoxic conditions, such as the isocitrate dehydrogenase, were previously reported [50]. By contrast, we observed a statistically significant upregulation of the isocitrate dehydrogenase *Idh3g* isoform only after hypoxia/reoxygenation (Fig 4). Upregulation of the constituents of the complexes III, IV and V indicates higher oxidative phosphorylation rates in hypoxia and hypoxia/reoxygenation samples compared to normoxia. A possible explanation is that under low O_2_ conditions, upregulation of these components may reflect the attempt of the cells to ensure an efficient energy gain from the available O_2_, similarly to the patterns in mitochondria of rat brain that had been acclimated to hypoxia [51, 52].

## Conclusion

There is little doubt that the unusual hypoxia-tolerance of the hooded seal brain is the result of a variety of molecular and biochemical adaptations. While we previously showed marked differences in the steady-state mRNA expression of the hooded seal brain and those of the ferret and other terrestrial mammals [30], the knowledge on the molecular responses of the brain was limited and restricted to the expression analysis of few genes by qRT-PCR [26]. In large parts, the seal brain responds to the hypoxic challenge in a similar way as the brain of other mammals, which includes the upregulation of typical stress proteins like cytokines and immediate early genes [39, 53]. However, none of the genes involved in the energy metabolism were found to be regulated in the previous studies of the ischemic brain [39, 54, 55], suggesting that their regulation is a specific response of the seal brain to hypoxia and reoxygenation. The observed changes in the expression of genes involved in the energy metabolism can be most convincingly interpreted as a switch to anaerobic energy metabolism (Figs 4 and 5; S3 Table). This conclusion is supported by the upregulation of genes that code for glycolytic enzymes, the downregulation of the pyruvate dehydrogenase complex, and the upregulation of the monocarboxylate transporter *Mct4*, which indicates lactate efflux from the cells.

However, the current data provide only a glimpse into the adaptations that are required to help the seal brain to cope with the hypoxic periods during the dive. Firstly and as already mentioned, transcriptome studies do not allow the detection of changes that occur on the posttranscriptional level. Secondly, although the brain slice technique is well established in animal models [24, 56, 57], it is unknown whether the responses in the slices, in fact, reflect the situation *in vivo*. Thirdly, our data only reflect the global changes and do not allow discriminating between the expression of genes in neurons and glia cells; in fact, there is ample immunohistological evidence that changes in the interplay between neurons and astrocytes is an important component in the adaptation of the hooded seal brain to hypoxia [27–29]. Cell-specific transcriptomes would be required to study cell-specific responses, but are currently difficult to obtain from brain tissue of the hooded seal.

## Supporting Information

S1 Fig Experimental design and time sequence of treatment. (A) Normoxia experiments. B. Hypoxia experiments. C. Reoxygenation experiments.
(PDF)

S2 Fig Comparison of the annotations of the two *de novo* assemblies of the hooded seal transcriptomes. The best blastx hit from the human RefSeq database for each contig was used in the analyses.
(PDF)

S3 Fig Correlation of untreated visual cortex with seal brain slice gene expression. (A) PCA of all transcripts (9347 features with RPKM >1) from the visual cortex (red), brain slices at normoxia (green), hypoxia (blue) and reoxygenation (yellow). (B) PCA of the statistically DE transcripts shared between all samples (109 features). A very similar transcriptomic response of the normoxia brain slices and the visual cortex versus hypoxia and reoxygenation brain slices is indicated.
(PDF)

S4 Fig Gene ontology in the domain "biological process". GO analysis was carried out for the significantly up- (A) and down- (B) regulated genes in the hooded seal brain slices after 1 h hypoxia (black) and after 1 h hypoxia followed by 20 min reoxygenation (white).
(PDF)

S5 Fig Gene ontology in the domain "protein class". GO analysis was carried out for the significantly up- (A) and down- (B) regulated genes in the hooded seal brain slices after 1 h hypoxia (black) and after 1 h hypoxia followed by 20 min reoxygenation (white).
(PDF)

S6 Fig Overview of the cellular energy metabolism of mammals. (PDF)

S7 Fig Correlation analyses of qRT-PCR and RNAseq data. The mRNA levels of *Pdha1* (A) and *Dlat* (B) were estimaned by RNAseq (RPKM, x-axis) and by qRT-PCR (y-axis).
(PDF)

S1 File Gene expression in brain slices from the visual cortex of the hooded seal. A. Gene expression (RPKM) of the brain slices at normoxia (n2-n4), hypoxia (h2-4) and after reoxygenation (r2-r4). A list with the expression levels of the genes (RPKM) from the untreated visual cortex is provided [30]. B. DGE test of slices kept under hypoxia vs. normoxia. C. DGE test of slices after reoxygenation vs. normoxia.
(XLSX)

S1 Table Hypoxia-regulated genes in the hooded seal brain. Significantly up- (A) and downregulated (B) genes in seal brain slices after 1 h hypoxia (N = 3 each).
(DOC)

S2 Table Hypoxia/reoxygenation-regulated genes in the hooded seal brain. Significantly up- (A) and downregulated (B) genes in seal brain slices after 1 h hypoxia followed by 20 min reoxygenation (N = 3 each).
(DOC)

S3 Table List of enzymes and other genes involved in the energy metabolism. The ENSEMBL identifiers of the ferret genome are given.
(DOC)

## References

[1] Dirnagl U, Iadecola C, Moskowitz MA. Pathobiology of ischaemic stroke: an integrated view. Trends Neurosci. 1999;22(9):391–7. 10441299 10.1016/s0166-2236(99)01401-0

[2] Chamorro A, Dirnagl U, Urra X, Planas AM. Neuroprotection in acute stroke: targeting excitotoxicity, oxidative and nitrosative stress, and inflammation. Lancet Neurol. 2016:in press. 10.1016/S1474-4422(16)00114-9 27180033

[3] Haddad GG, Jiang C. O_2_ deprivation in the central nervous system: on mechanisms of neuronal response, differential sensitivity and injury. Prog Neurobiol. 1993;40(3):277–318. 7680137 10.1016/0301-0082(93)90014-j

[4] Hansen AJ. Effect of anoxia on ion distribution in the brain. Physiol Rev. 1985;65(1):101–48. 3880896 10.1152/physrev.1985.65.1.101

[5] Larson J, Drew KL, Folkow LP, Milton SL, Park TJ. No oxygen? No problem! Intrinsic brain tolerance to hypoxia in vertebrates. J Exp Biol. 2014;217(Pt 7):1024–39. 10.1242/jeb.085381 24671961 PMC3966918

[6] Nathaniel TI, Williams-Hernandez A, Hunter AL, Liddy C, Peffley DM, Umesiri FE, et al Tissue hypoxia during ischemic stroke: adaptive clues from hypoxia-tolerant animal models. Brain Res Bull. 2015;114:1–12. 10.1016/j.brainresbull.2015.02.006 25738761

[7] Bano D, Nicotera P. Ca^2+^ signals and neuronal death in brain ischemia. Stroke. 2007;38(2 Suppl):674–6. 10.1161/01.STR.0000256294.46009.29 17261713

[8] Therade-Matharan S, Laemmel E, Carpentier S, Obata Y, Levade T, Duranteau J, et al Reactive oxygen species production by mitochondria in endothelial cells exposed to reoxygenation after hypoxia and glucose depletion is mediated by ceramide. Am J Physiol Regul Integr Comp Physiol. 2005;289(6):R1756–62. 10.1152/ajpregu.00480.2004 16278342

[9] Kerem D, Elsner R. Cerebral tolerance to asphyxial hypoxia in the harbor seal. Respir Physiol. 1973;19(2):188–200. 4763083 10.1016/0034-5687(73)90077-7

[10] Butler PJ. Metabolic regulation in diving birds and mammals. Respir Physiol Neurobiol. 2004;141(3):297–315. 10.1016/j.resp.2004.01.010 15288601

[11] Butler PJ, Jones DR. Physiology of diving of birds and mammals. Physiol Rev. 1997;77(3):837–99. 9234967 10.1152/physrev.1997.77.3.837

[12] Ramirez JM, Folkow LP, Blix AS. Hypoxia tolerance in mammals and birds: from the wilderness to the clinic. Annu Rev Physiol. 2007;69:113–43. 10.1146/annurev.physiol.69.031905.163111 17037981

[13] Scholander PF. Experimantal investigations on the respiratory function in diving mammals and birds. Hvalradets Skr. 1940;22:1–131.

[14] Zapol WM, Liggins GC, Schneider RC, Qvist J, Snider MT, Creasy RK, et al Regional blood flow during simulated diving in the conscious Weddell seal. J Appl Physiol Respir Environ Exerc Physiol. 1979;47(5):968–73. 511722 10.1152/jappl.1979.47.5.968

[15] Qvist J, Hill RD, Schneider RC, Falke KJ, Liggins GC, Guppy M, et al Hemoglobin concentrations and blood gas tensions of free-diving Weddell seals. J Appl Physiol. 1986;61(4):1560–9. 3096941 10.1152/jappl.1986.61.4.1560

[16] Meir JU, Champagne CD, Costa DP, Williams CL, Ponganis PJ. Extreme hypoxemic tolerance and blood oxygen depletion in diving elephant seals. Am J Physiol Regul Integr Comp Physiol. 2009;297(4):R927–39. 10.1152/ajpregu.00247.2009 19641132

[17] Williams TM, Zavanelli M, Miller MA, Goldbeck RA, Morledge M, Casper D, et al Running, swimming and diving modifies neuroprotecting globins in the mammalian brain. Proc R Soc Lond, Ser B: Biol Sci. 2008;275(1636):751–8. 10.1098/rspb.2007.1484 PMC2596902 18089537

[18] Davis RW. A review of the multi-level adaptations for maximizing aerobic dive duration in marine mammals: from biochemistry to behavior. J Comp Physiol [B]. 2014;184(1):23–53. 10.1007/s00360-013-0782-z 24126963

[19] Blix AS, Folkow B. Cardiovascular adjustments to diving in mammals and birds In: Sheperd JT, Abboud FM, editors. Handbook of Physiology—The Cardiovascular System III. Bethesda: Am. Physiol. Soc.; 1983 p. 917–45.

[20] Ponganis PJ. Diving Mammals. Comp Physiol. 2011;1(1):447–65. 10.1002/cphy.c091003 23737181

[21] Blix AS, Elsner R, Kjekshus JK. Cardiac output and its distribution through capillaries and A-V shunts in diving seals. Acta Physiol Scand. 1983;118(2):109–16. 10.1111/j.1748-1716.1983.tb07250.x 6624500

[22] Folkow LP, Blix AS. Air breathers under water: diving mammals and birds In: Nilsson GE, editor. Respiratory physiology of vertebrates Life with and without oxygen. Cambridge: Cambridge University Press; 2010 p. 222–64.

[23] Folkow LP, Blix AS. Diving behaviour of hooded seals (*Cystophora cristata*) in the Greenland and Norwegian Seas. Polar Biol. 1999;22(1):61–74.

[24] Folkow LP, Ramirez JM, Ludvigsen S, Ramirez N, Blix AS. Remarkable neuronal hypoxia tolerance in the deep-diving adult hooded seal (*Cystophora cristata*). Neurosci Lett. 2008;446(2–3):147–50. 10.1016/j.neulet.2008.09.040 18824079

[25] Ramirez JM, Folkow LP, Ludvigsen S, Ramirez PN, Blix AS. Slow intrinsic oscillations in thick neocortical slices of hypoxia tolerant deep diving seals. Neuroscience. 2011;177:35–42. 10.1016/j.neuroscience.2010.12.032 21185914

[26] Czech-Damal NU, Geiseler SJ, Hoff ML, Schliep R, Ramirez JM, Folkow LP, et al The role of glycogen, glucose and lactate in neuronal activity during hypoxia in the hooded seal (*Cystophora cristata*) brain. Neuroscience. 2014;275:374–83. 10.1016/j.neuroscience.2014.06.024 24959743

[27] Mitz SA, Reuss S, Folkow LP, Blix AS, Ramirez JM, Hankeln T, et al When the brain goes diving: glial oxidative metabolism may confer hypoxia tolerance to the seal brain. Neuroscience. 2009;163(2):552–60. 10.1016/j.neuroscience.2009.06.058 19576963

[28] Schneuer M, Flachsbarth S, Czech-Damal NU, Folkow LP, Siebert U, Burmester T. Neuroglobin of seals and whales: evidence for a divergent role in the diving brain. Neuroscience. 2012;223:35–44. 10.1016/j.neuroscience.2012.07.052 22864183

[29] Hoff MLM, Fabrizius A, Folkow LP, Burmester T. An atypical distribution of lactate dehydrogenase isoenzymes in the hooded seal (*Cystophora cristata*) brain may reflect a biochemical adaptation to diving. J Comp Physiol [B]. 2016;186(3):373–86. 10.1007/s00360-015-0956-y 26820264

[30] Fabrizius A, Hoff MLM, Engler G, Folkow LP, Burmester T. When the brain goes diving: transcriptome analysis reveals a reduced aerobic energy metabolism and increased stress proteins in the seal brain. BMC Genomics. 2016;17:583 10.1186/s12864-016-2892-y 27507242 PMC4979143

[31] Blix AS, Walløe L, Messelt EB, Folkow LP. Selective brain cooling and its vascular basis in diving seals. J Exp Biol. 2010;213(Pt 15):2610–6. 10.1242/jeb.040345 20639422

[32] Odden A, Folkow LP, Caputa M, Hotvedt R, Blix AS. Brain cooling in diving seals. Acta Physiol Scand. 1999;166(1):77–8. 10.1046/j.1365-201x.1999.00536.x 10372982

[33] Murphy B, Zapol WM, Hochachka PW. Metabolic activities of heart, lung, and brain during diving and recovery in the Weddell seal. J Appl Physiol Respir Environ Exerc Physiol. 1980;48(4):596–605. 7380685 10.1152/jappl.1980.48.4.596

[34] Geiseler SJ, Larson J, Folkow LP. Synaptic transmission despite severe hypoxia in hippocampal slices of the deep-diving hooded seal. Neuroscience. 2016;334:39–46. 10.1016/j.neuroscience.2016.07.034 27480049

[35] Foote AD, Liu Y, Thomas GW, Vinar T, Alfoldi J, Deng J, et al Convergent evolution of the genomes of marine mammals. Nat Genet. 2015;47(3):272–5. 10.1038/ng.3198 25621460 PMC4644735

[36] Peng X, Alfoldi J, Gori K, Eisfeld AJ, Tyler SR, Tisoncik-Go J, et al The draft genome sequence of the ferret (*Mustela putorius furo*) facilitates study of human respiratory disease. Nat Biotechnol. 2014;32(12):1250–5. 10.1038/nbt.3079 25402615 PMC4262547

[37] Mi HY, Muruganujan A, Casagrande JT, Thomas PD. Large-scale gene function analysis with the PANTHER classification system. Nat Protoc. 2013;8(8):1551–66. 10.1038/nprot.2013.092 23868073 PMC6519453

[38] Robinson MD, Smyth GK. Small-sample estimation of negative binomial dispersion, with applications to SAGE data. Biostatistics. 2008;9(2):321–32. 10.1093/biostatistics/kxm030 17728317

[39] Büttner F, Cordes C, Gerlach F, Heimann A, Alessandri B, Luxemburger U, et al Genomic response of the rat brain to global ischemia and reperfusion. Brain Res. 2009;1252:1–14. 10.1016/j.brainres.2008.10.045 19071098

[40] Barone FC, Feuerstein GZ. Inflammatory mediators and stroke: new opportunities for novel therapeutics. J Cereb Blood Flow Metab. 1999;19(8):819–34. 10.1097/00004647-199908000-00001 10458589

[41] Yi JH, Park SW, Kapadia R, Vemuganti R. Role of transcription factors in mediating post-ischemic cerebral inflammation and brain damage. Neurochem Int. 2007;50(7–8):1014–27. 10.1016/j.neuint.2007.04.019 17532542 PMC2040388

[42] Danton GH, Dietrich WD. Inflammatory mechanisms after ischemia and stroke. J Neuropathol Exp Neurol. 2003;62(2):127–36. 12578222 10.1093/jnen/62.2.127

[43] Berg JM, Tymoczko JL, Stryer L. Biochemistry. 7th edition ed. New York: W. H. Freeman; 2012 1054 p.

[44] Simpson IA, Carruthers A, Vannucci SJ. Supply and demand in cerebral energy metabolism: the role of nutrient transporters. J Cereb Blood Flow Metab. 2007;27(11):1766–91. 10.1038/sj.jcbfm.9600521 17579656 PMC2094104

[45] Gao C, Wang C, Liu B, Wu H, Yang Q, Jin J, et al Intermittent hypoxia preconditioning-induced epileptic tolerance by upregulation of monocarboxylate transporter 4 expression in rat hippocampal astrocytes. Neurochem Res. 2014;39(11):2160–9. 10.1007/s11064-014-1411-2 25146899

[46] Gao C, Zhu W, Tian L, Zhang J, Li Z. MCT4-mediated expression of EAAT1 is involved in the resistance to hypoxia injury in astrocyte-neuron co-cultures. Neurochem Res. 2015;40(4):818–28. 10.1007/s11064-015-1532-2 25645447

[47] Liu B, Niu L, Shen MZ, Gao L, Wang C, Li J, et al Decreased astroglial monocarboxylate transporter 4 expression in temporal lobe epilepsy. Mol Neurobiol. 2014;50(2):327–38. 10.1007/s12035-013-8619-z 24464262

[48] Kikuchi D, Minamishima YA, Nakayama K. Prolyl-hydroxylase PHD3 interacts with pyruvate dehydrogenase (PDH)-E1beta and regulates the cellular PDH activity. Biochem Biophys Res Commun. 2014;451(2):288–94. 10.1016/j.bbrc.2014.07.114 25088999

[49] Kim JW, Tchernyshyov I, Semenza GL, Dang CV. HIF-1-mediated expression of pyruvate dehydrogenase kinase: a metabolic switch required for cellular adaptation to hypoxia. Cell Metab. 2006;3(3):177–85. 10.1016/j.cmet.2006.02.002 16517405

[50] Murphy BJ, Robin ED, Tapper DP, Wong RJ, Clayton DA. Hypoxic coordinate regulation of mitochondrial enzymes in mammalian cells. Science. 1984;223(4637):707–9. 6320368 10.1126/science.6320368

[51] Luk’yanova LD, Kirova YI. Mitochondria-controlled signaling mechanisms of brain protection in hypoxia. Front Neurosci. 2015;9:320 10.3389/fnins.2015.00320 26483619 PMC4589588

[52] Luk’yanova LD, Chernobaeva GN, Romanova VE. Effects of adaptation to intermittent hypoxia on oxidative phosphorylation in brain mitochondria of rats with different sensitivities toward oxygen deficiency. Bull Exp Biol Med. 1995;120:1189–92.

[53] Bonestroo HJ, Nijboer CH, van Velthoven CT, Kavelaars A, Hack CE, van Bel F, et al Cerebral and hepatic inflammatory response after neonatal hypoxia-ischemia in newborn rats. Dev Neurosci. 2013;35(2–3):197–211. 10.1159/000346685 23689428

[54] Jin K, Mao XO, Eshoo MW, Nagayama T, Minami M, Simon RP, et al Microarray analysis of hippocampal gene expression in global cerebral ischemia. Ann Neurol. 2001;50(1):93–103. 11456315 10.1002/ana.1073

[55] Schmidt-Kastner R, Zhang B, Belayev L, Khoutorova L, Amin R, Busto R, et al DNA microarray analysis of cortical gene expression during early recirculation after focal brain ischemia in rat. Brain Res Mol Brain Res. 2002;108(1–2):81–93. 12480181 10.1016/s0169-328x(02)00516-8

[56] Lonchamp E, Dupont JL, Beekenkamp H, Poulain B, Bossu JL. The mouse cerebellar cortex in organotypic slice cultures: an *in vitro* model to analyze the consequences of mutations and pathologies on neuronal survival, development, and function. Crit Rev Neurobiol. 2006;18(1–2):179–86. 17725520 10.1615/critrevneurobiol.v18.i1-2.180

[57] Shibuya I, Kabashima N, Ibrahim N, Setiadji SV, Ueta Y, Yamashita H. Pre- and postsynaptic modulation of the electrical activity of rat supraoptic neurones. Exp Physiol. 2000;85 Spec No:145S–51S. 10795917 10.1111/j.1469-445x.2000.tb00018.x

