# Supplementary material for: Transcriptome Analysis Identifies Key Metabolic Changes in the Hooded Seal (Cystophora cristata) Brain in Response to Hypoxia and Reoxygenation
Source: PLoS One. 2017 Jan 3;12(1):e0169366. doi: 10.1371/journal.pone.0169366 (PMC5207758; doi:10.1371/journal.pone.0169366)
Supplement: S1 Fig — (A) Normoxia experiments. B. Hypoxia experiments. C. Reoxygenation experiments. (PDF) [file pone.0169366.s001.pdf]

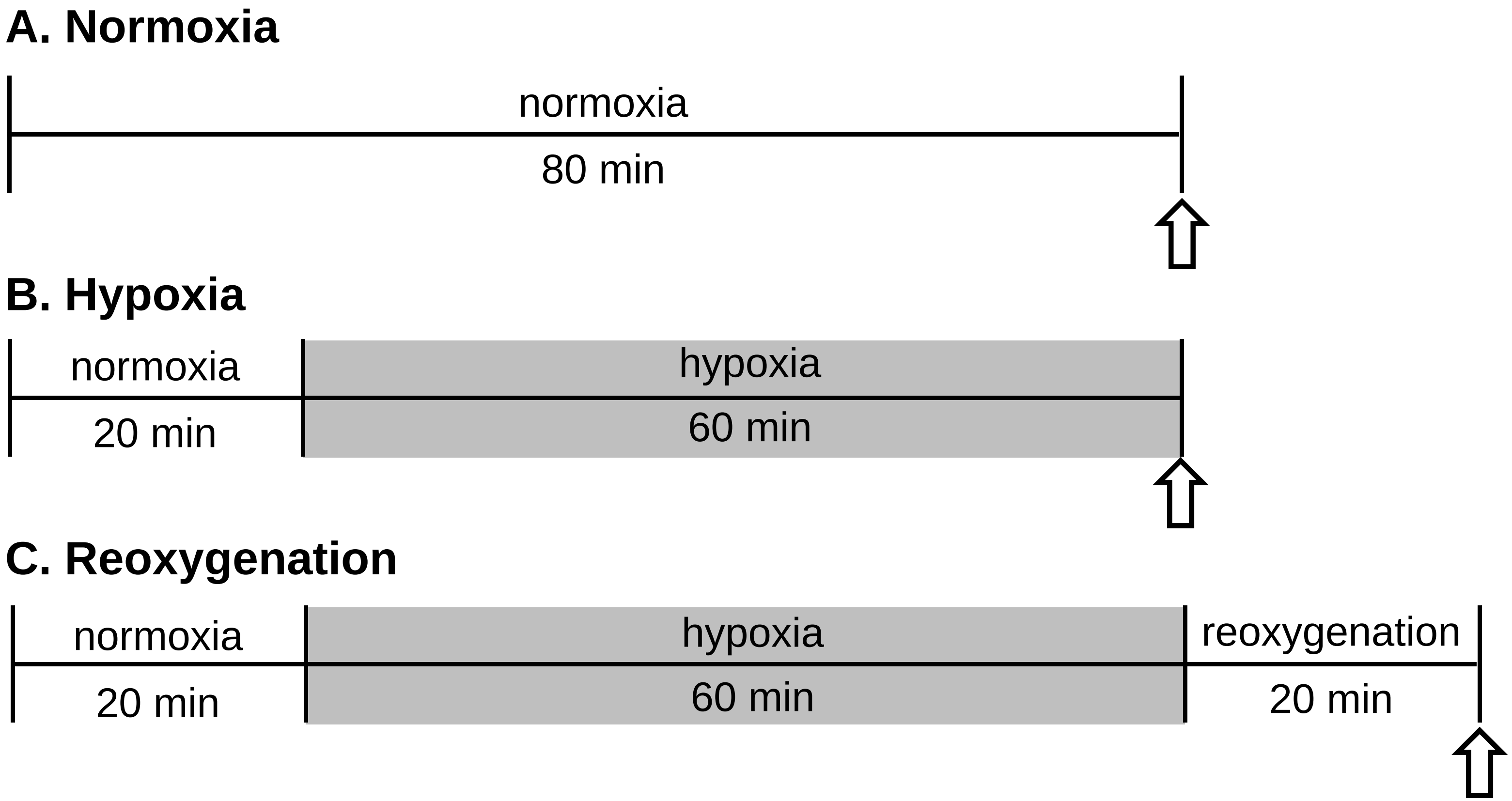

S1 Figure. Experimental design and time sequence of treatment. (A) Normoxia experiments. B. Hypoxia experiments. C. Reoxygenation experiments.
