# Supplementary material for: Transcriptome Analysis Identifies Key Metabolic Changes in the Hooded Seal (Cystophora cristata) Brain in Response to Hypoxia and Reoxygenation
Source: PLoS One. 2017 Jan 3;12(1):e0169366. doi: 10.1371/journal.pone.0169366 (PMC5207758; doi:10.1371/journal.pone.0169366)
Supplement: S2 Fig — The best blastx hit from the human RefSeq database for each contig was used in the analyses. (PDF) [file pone.0169366.s002.pdf]

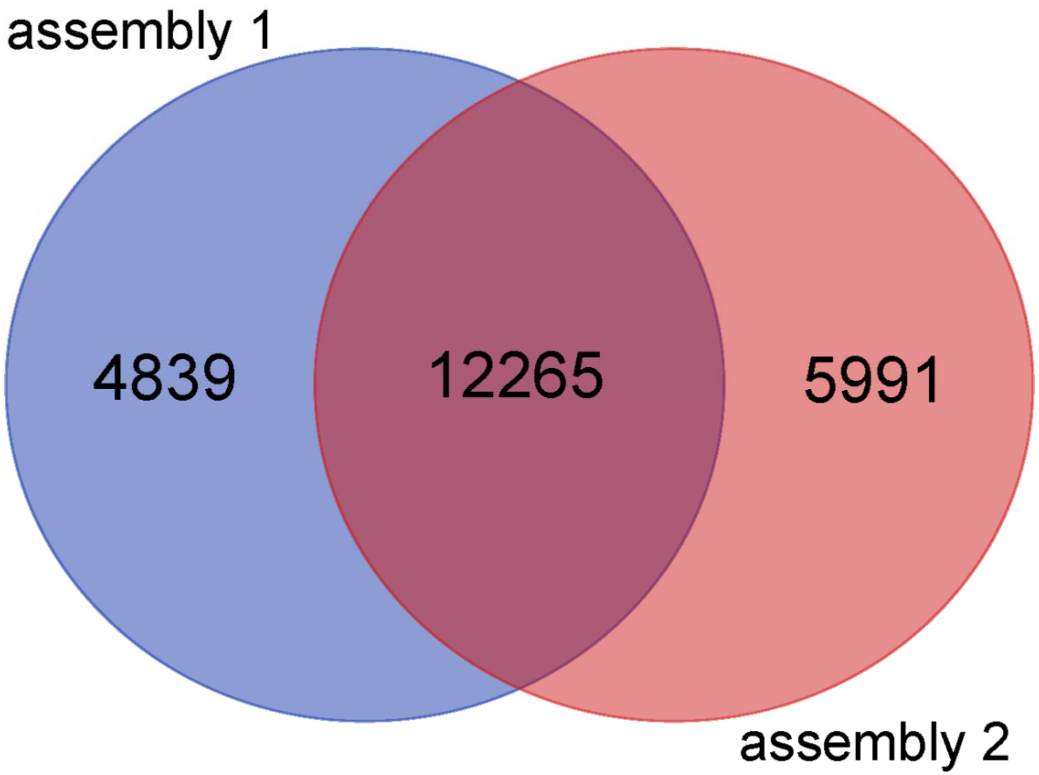

S2 Figure. Comparison of the annotations of the two de novo assemblies of the hooded seal transcriptomes.
The best blastx hit from the human RefSeq database for each contig was used in the analyses.
