# Supplementary material for: Transcriptome Analysis Identifies Key Metabolic Changes in the Hooded Seal (Cystophora cristata) Brain in Response to Hypoxia and Reoxygenation
Source: PLoS One. 2017 Jan 3;12(1):e0169366. doi: 10.1371/journal.pone.0169366 (PMC5207758; doi:10.1371/journal.pone.0169366)
Supplement: S3 Fig — (A) PCA of all transcripts (9347 features with RPKM >1) from the visual cortex (red), brain slices at normoxia (green), hypoxia (blue) and reoxygenation (yellow). (B) PCA of the statistically DE transcripts shared between all samples (109 features). A very similar transcriptomic response of the normoxia brain slices and the visual cortex versus hypoxia and reoxygenation brain slices is indicated. (PDF) [file pone.0169366.s003.pdf]

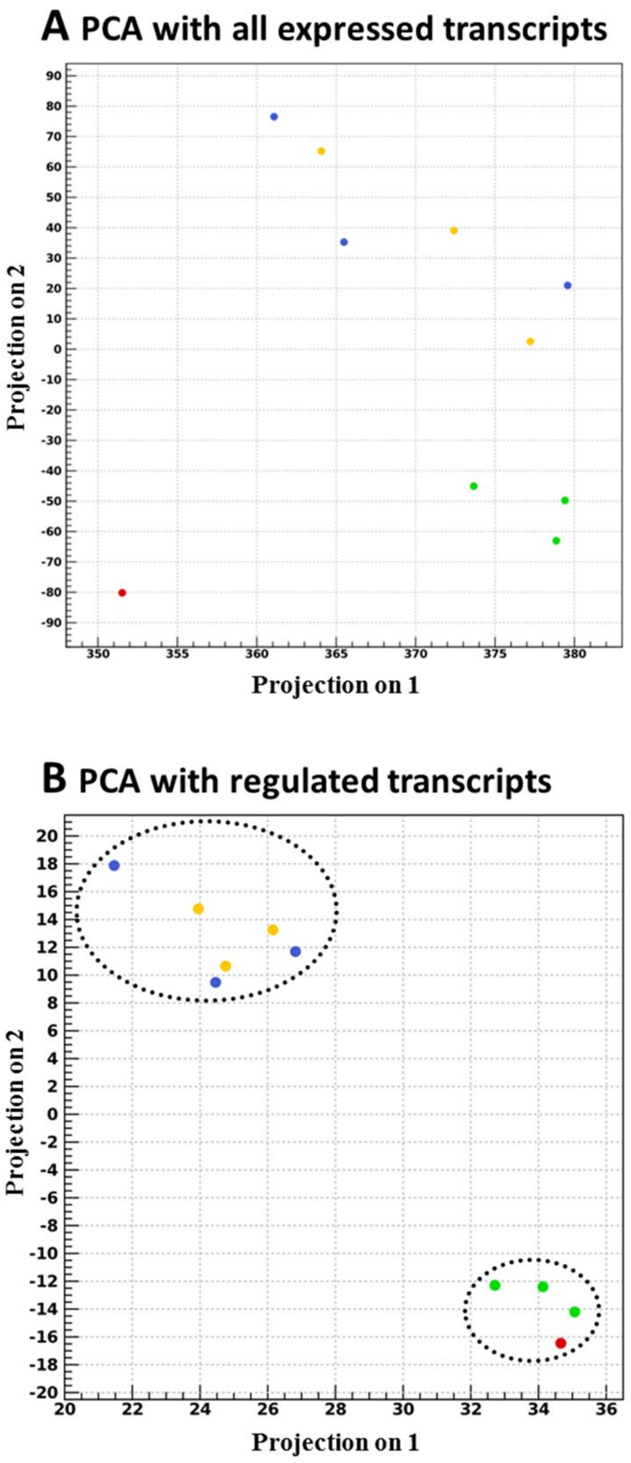

S3 Figure. Correlation of untreated visual cortex with seal brain slice gene expression. (A) PCA of all transcripts (9347 features with RPKM >1) from the visual cortex (red), brain slices at normoxia (green), hypoxia (blue) and reoxygenation (yellow). (B) PCA of the statistically DE transcripts shared between all samples (109 features). A very similar transcriptomic response of the normoxia brain slices and the visual cortex versus hypoxia and reoxygenation brain slices is indicated.
