# Supplementary material for: Transcriptome Analysis Identifies Key Metabolic Changes in the Hooded Seal (Cystophora cristata) Brain in Response to Hypoxia and Reoxygenation
Source: PLoS One. 2017 Jan 3;12(1):e0169366. doi: 10.1371/journal.pone.0169366 (PMC5207758; doi:10.1371/journal.pone.0169366)
Supplement: S4 Fig — GO analysis was carried out for the significantly up- (A) and down- (B) regulated genes in the hooded seal brain slices after 1 h hypoxia (black) and after 1 h hypoxia followed by 20 min reoxygenation (white). (PDF) [file pone.0169366.s004.pdf]

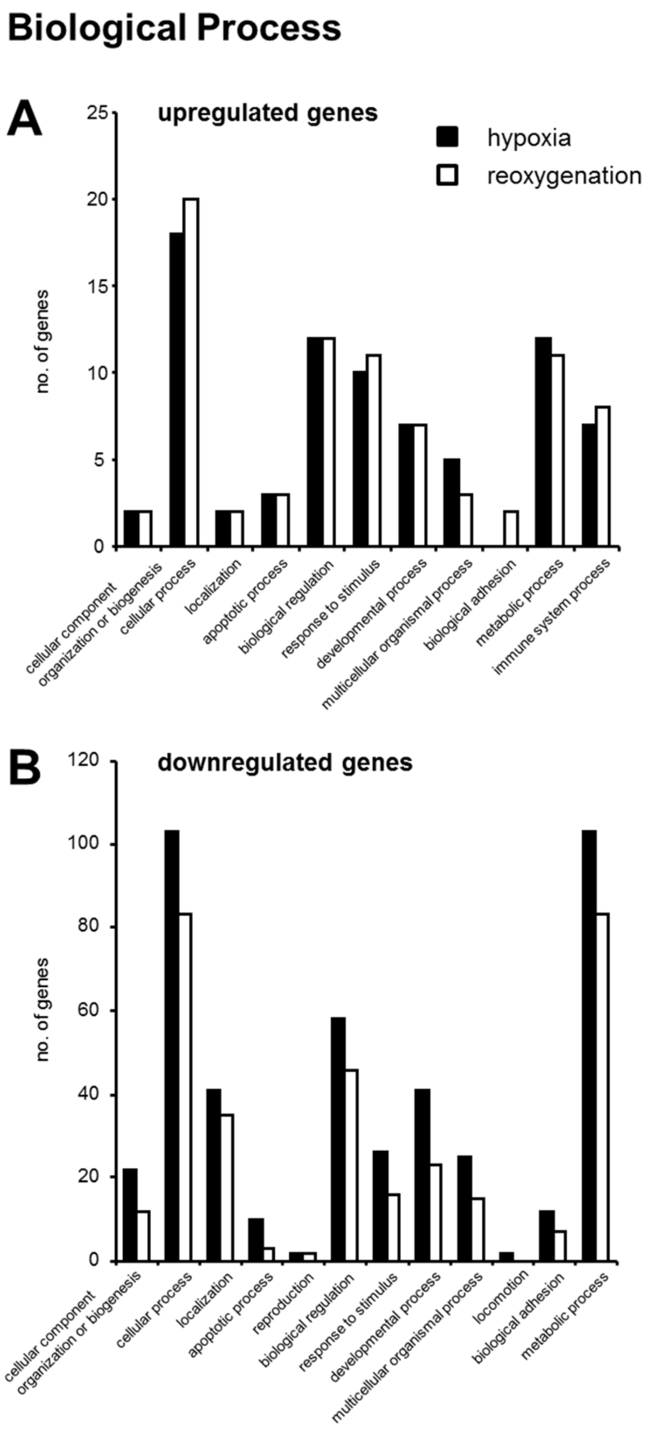

S4 Figure. Gene ontology in the domain "biological process". GO analysis was carried out for the significantly up- (A) and down- (B) regulated genes in the hooded seal brain slices after 1 h hypoxia (black) and after 1 h hypoxia followed by 20 min reoxygenation (white).
