# Supplementary figures and images for: Transcriptome Analysis Identifies Key Metabolic Changes in the Hooded Seal (Cystophora cristata) Brain in Response to Hypoxia and Reoxygenation
Source: PLoS One. 2017 Jan 3;12(1):e0169366. doi: 10.1371/journal.pone.0169366 (PMC5207758; doi:10.1371/journal.pone.0169366)

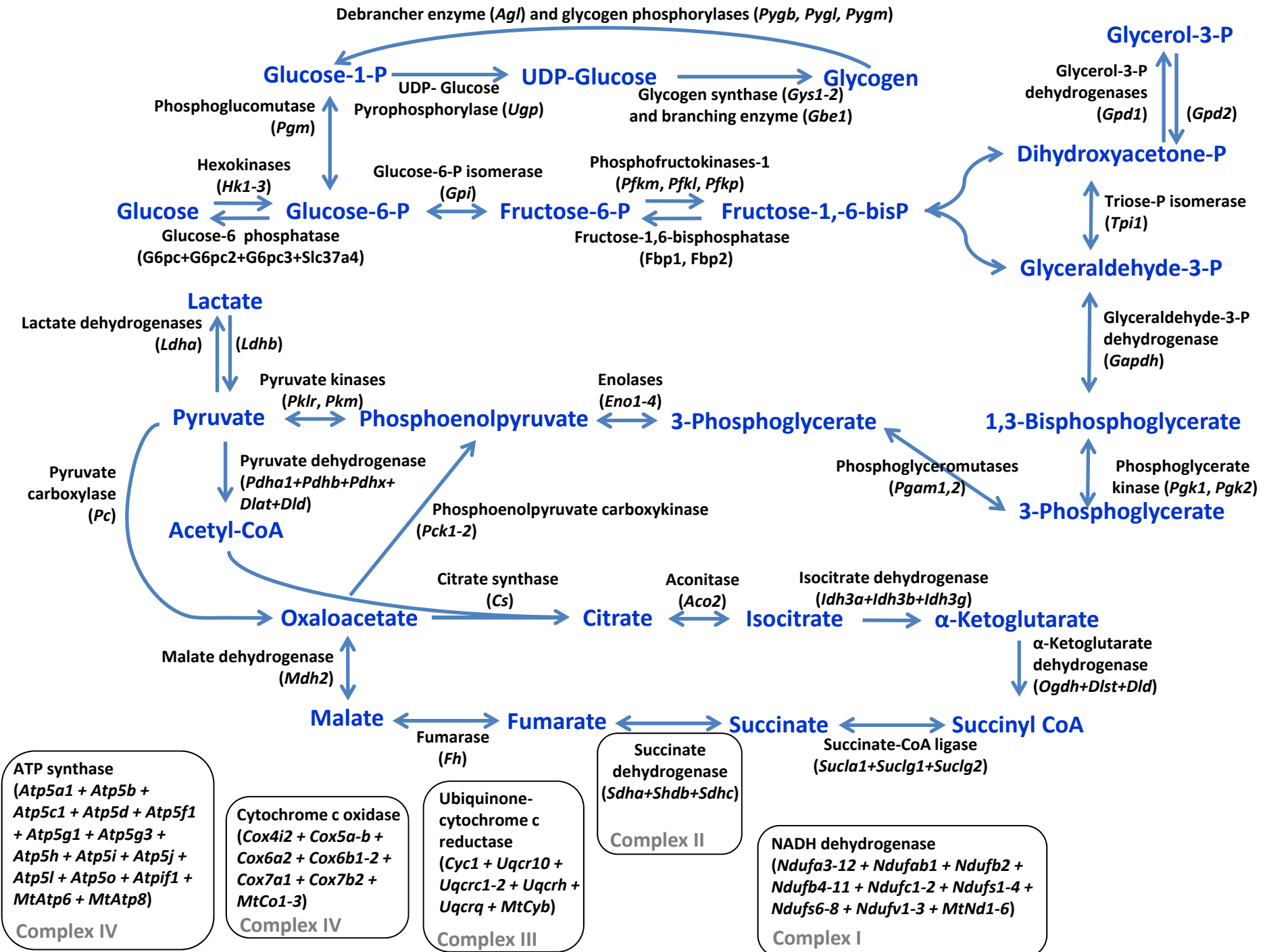

Supplement: S6 Fig — (PDF) [file pone.0169366.s006.pdf]
