# Supplementary material for: Transcriptome Analysis Identifies Key Metabolic Changes in the Hooded Seal (Cystophora cristata) Brain in Response to Hypoxia and Reoxygenation
Source: PLoS One. 2017 Jan 3;12(1):e0169366. doi: 10.1371/journal.pone.0169366 (PMC5207758; doi:10.1371/journal.pone.0169366)
Supplement: S7 Fig — The mRNA levels of Pdha1 (A) and Dlat (B) were estimaned by RNAseq (RPKM, x-axis) and by qRT-PCR (y-axis). (PDF) [file pone.0169366.s007.pdf]

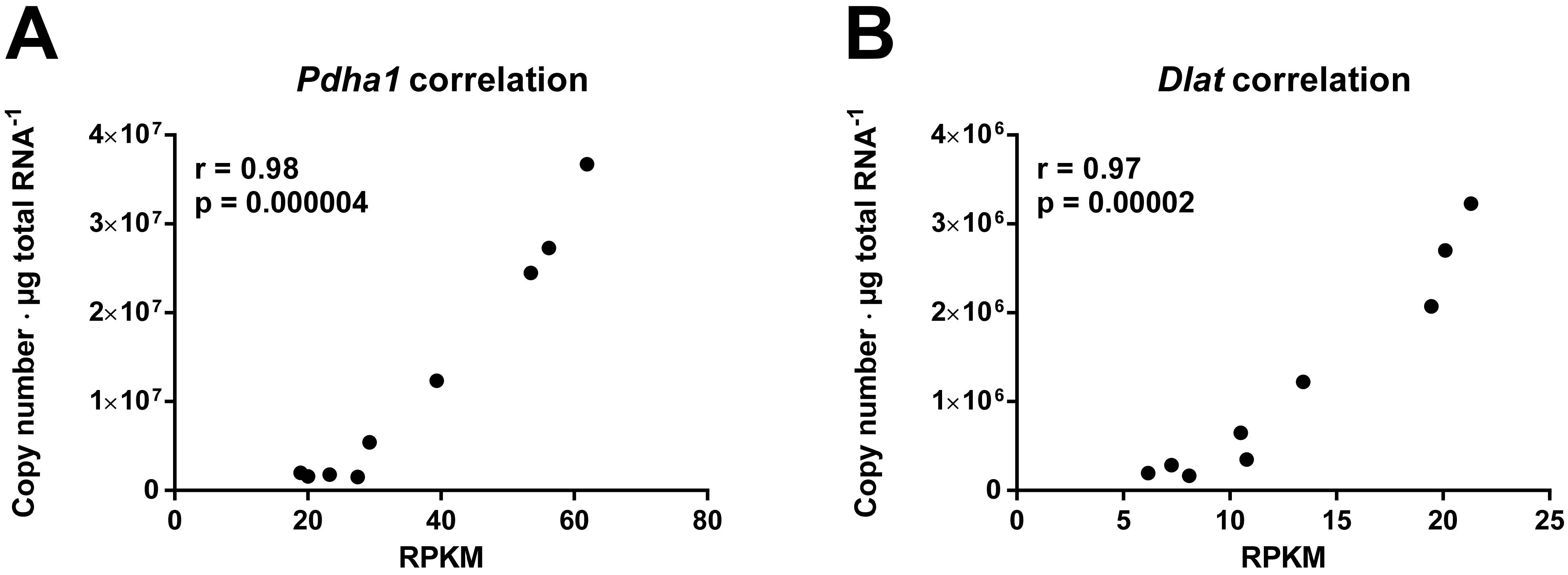

S7 Figure. Correlation analyses of qRT-PCR and RNAseq data. The mRNA levels of *Pdha1* (A) and *Dlat* (B) were estimaned by RNAseq (RPKM, x-axis) and by qRT-PCR (y-axis).
