# Supplementary material for: Transcriptome Analysis Identifies Key Metabolic Changes in the Hooded Seal (Cystophora cristata) Brain in Response to Hypoxia and Reoxygenation
Source: PLoS One. 2017 Jan 3;12(1):e0169366. doi: 10.1371/journal.pone.0169366 (PMC5207758; doi:10.1371/journal.pone.0169366)
Supplement: S1 Table — Significantly up- (A) and downregulated (B) genes in seal brain slices after 1 h hypoxia (N = 3 each). (DOC) [file pone.0169366.s009.doc]

**S1 Table.** Significantly up- (A) and downregulated (B) genes in seal brain slices after 1 h hypoxia (N = 3 each).

| **A. Upregulated genes** | **Gene symbol** | **Fold change** |
| --- | --- | --- |
| Interleukin-1β | Il1b | 668.85 |
| chemokine (C-C motif) ligand 3 | Ccl3 | 65.23 |
| chemokine (C-C motif) ligand 4 | Ccl4 | 55.42 |
| v-maf avian musculoaponeurotic fibrosarcoma oncogene homolog F | Maff | 45.82 |
| interleukin 1 receptor antagonist | Il1rn | 36.92 |
| activating transcription factor 3 | Atf3 | 35.98 |
| growth-regulated alpha protein-like | Cxcl1 | 26.55 |
| plasminogen activator, urokinase receptor | Plaur | 18.47 |
| Shisa family member 8 | Shisa8 | 15.57 |
| FBJ murine osteosarcoma viral oncogene homolog B | Fosb | 12.61 |
| zinc finger CCCH-type containing 12A | Zc3h12a | 10.24 |
| ZFP36 ring finger protein | Zfp36 | 10.09 |
| chemokine (C-C motif) ligand 2 | Ccl2 | 9.91 |
| solute carrier family 1 (neutral amino acid transporter), member 5 | Slc1a5 | 9.63 |
| growth arrest and DNA-damage-inducible, gamma | Gadd45g | 7.35 |
| growth arrest and DNA-damage-inducible, gamma interacting protein | Gadd45gip1 | 7.15 |
| transgelin 2 | Tagln2 | 6.65 |
| adrenomedullin | Adm | 5.9 |
| BTG family, member 2 | Btg2 | 5.8 |
| regulator of G-protein signaling 1 | Rgs1 | 5.77 |
| protein phosphatase 1, regulatory subunit 15A | Ppp1r15a | 5.43 |
| ribonuclease/angiogenin inhibitor 1 | Rnh1 | 5.38 |
| protein QIL-1 | Qil1 | 5.36 |
| archaelysin family metallopeptidase 1 | Amz1 | 5.27 |
| growth arrest and DNA-damage-inducible, beta | Gadd45b | 4.93 |
| apolipoprotein A-I | Apoa1 | 4.47 |
| arrestin domain containing 2 | Arrdc2 | 4.39 |
| lamin A/C | Lmna | 4.29 |
| lymphocyte-specific protein 1 | Lsp1 | 4.23 |
| aarF domain containing kinase 5 | Adck5 | 3.96 |
| nuclear receptor subfamily 4, group A, member 1 | Nr4a1 | 3.94 |
| B-cell CLL/lymphoma 3 | Bcl3 | 3.9 |
| Ets2 repressor factor | Erf | 3.73 |
| dual specificity phosphatase 1 | Dusp1 | 3.52 |

| **B. Downregulated genes** | **Gene symbol** | **Fold change** |
| --- | --- | --- |
| zinc finger protein 211 | Znf211 | -113.94 |
| stonin 2 | Ston2 | -28.17 |
| zinc finger protein 425 | Znf425 | -11.46 |
| solute carrier family 4 (sodium bicarbonate cotransporter), member 4 | Slc4a4 | -7.95 |
| sacsin molecular chaperone | Sacs | -7.9 |
| [dmX-like protein 1](http://blast.ncbi.nlm.nih.gov/Blast.cgi#alnHdr_511878396) | Dmxl1 | -7.74 |
| kelch-like family member 11 | Klhl11 | -7.12 |
| low density lipoprotein receptor-related protein 5-like | [Lrp5](http://omim.org/entry/603506?search=low density lipoprotein receptor-related protein 5-like &highlight=like density receptorrelated 5like lipoprotein low proteinaceous protein) | -6.69 |
| v-erb-b2 avian erythroblastic leukemia viral oncogene homolog 4 | Erbb4 | -6.64 |
| pleckstrin homology domain containing, family H (with MyTH4 domain) member 2 | Plekhh2 | -6.32 |
| activin A receptor, type IC | Acvr1c | -6.11 |
| clock circadian regulator | Clock | -5.88 |
| glutamine and serine rich 1 | Qser1 | -5.84 |
| mitogen-activated protein kinase kinase kinase 2 | Map3k2 | -5.75 |
| RAR-related orphan receptor B | Rorb | -5.61 |
| solute carrier family 7 (anionic amino acid transporter light chain, xc-system), member 11 | Slc7a11 | -5.6 |
| Caveolin-2 | Cav2 | -5.54 |
| neuronal tyrosine-phosphorylated phosphoinositide-3-kinase adaptor 2 | Nyap2 | -5.48 |
| solute carrier family 1 (glial high affinity glutamate transporter), member 2 | Slc1a2 | -5.41 |
| ATPase type 13A4 | Atp13a4 | -5.29 |
| DCC netrin 1 receptor | Dcc | -5.16 |
| paternally expressed 3 | Peg3 | -5.16 |
| potassium voltage-gated channel, subfamily H (eag-related), member 7 | Kcnh7 | -5.13 |
| ubiquitin specific peptidase 37 | Usp37 | -5.12 |
| family with sequence similarity 178, member A | Fam178a | -5.1 |
| teneurin transmembrane protein 1 | Tenm1 | -5.1 |
| SMG1 homolog, phosphatidylinositol 3-kinase-related kinase | Smg1 | -5.08 |
| UDP-glucose glycoprotein glucosyltransferase 1 | Uggt1 | -5.08 |
| unconventional myosin-IXa-like | [Myo9a](http://omim.org/entry/604875?search=myosin IXa&highlight=ixa myosin) | -5.06 |
| ATP-binding cassette, sub-family A (ABC1), member 1 | Abca1 | -5 |
| tau tubulin kinase 2 | Ttbk2 | -4.97 |
| baculoviral IAP repeat containing 8 | Xiap | -4.93 |
| zinc finger with KRAB and SCAN domains 1 | Zkscan1 | -4.93 |
| leucyl/cystinyl aminopeptidase | Lnpep | -4.92 |
| A kinase (PRKA) anchor protein 6 | Akap6 | -4.91 |
| contactin associated protein-like 5 | Cntnap5 | -4.85 |
| neurexin 1 | Nrxn1 | -4.85 |
| oligophrenin 1 | Ophn1 | -4.81 |
| TAO kinase 1 | Taok1 | -4.76 |
| chromodomain helicase DNA binding protein 9 | Chd9 | -4.75 |
| SET binding protein 1 | Setbp1 | -4.73 |
| transient receptor potential cation channel, subfamily M, member 3 | Trpm3 | -4.73 |
| transforming growth factor, beta receptor III | Tgfbr3 | -4.71 |
| dystonin | Dst | -4.7 |
| leukemia inhibitory factor receptor alpha | Lifr | -4.7 |
| unc-5 homolog D | Unc5d | -4.64 |
| zinc finger with KRAB and SCAN domains 8 | Zkscan8 | -4.61 |
| ethanolaminephosphotransferase 1 (CDP-ethanolamine-specific) | Ept1 | -4.58 |
| G protein-coupled receptor 98 | Gpr98 | -4.57 |
| family with sequence similarity 126, member B | Fam126b | -4.55 |
| activating transcription factor 7 interacting protein | Atf7ip | -4.54 |
| OTU deubiquitinase 4 | Otud4 | -4.54 |
| microtubule-associated protein 1B | Map1b | -4.52 |
| zinc finger, matrin-type 3 | Zmat3 | -4.51 |
| contactin 3 (plasmacytoma associated) | Cntn3 | -4.5 |
| mannosidase, alpha, class 1A, member 2 | Man1a2 | -4.49 |
| solute carrier family 38, member 1 | Slc38a1 | -4.49 |
| ATPase, aminophospholipid transporter (APLT), class I, type 8A, member 1 | Atp8a1 | -4.46 |
| gamma-aminobutyric acid (GABA) A receptor, beta 1 | Gabrb1 | -4.44 |
| p21 protein (Cdc42/Rac)-activated kinase 3 | Pak3 | -4.42 |
| sodium channel, voltage-gated, type I, alpha subunit | Scn1a | -4.41 |
| thyroid hormone receptor, beta | Thrb | -4.41 |
| spectrin repeat containing, nuclear envelope 1 | Syne1 | -4.4 |
| dystrophin | Dmd | -4.34 |
| DENN/MADD domain containing 5B | Dennd5b | -4.32 |
| centrosomal protein 85kDa-like | Cep85l | -4.31 |
| protein tyrosine phosphatase, receptor type, D | Ptprd | -4.31 |
| RAB11 family interacting protein 2 (class I) | Rab11fip2 | -4.31 |
| metaxin 3 | Mtx3 | -4.3 |
| folliculin interacting protein 2 | Fnip2 | -4.28 |
| tripartite motif containing 33 | Trim33 | -4.28 |
| ryanodine receptor 2 (cardiac) | Ryr2 | -4.27 |
| AT rich interactive domain 2 (ARID, RFX-like) | Arid2 | -4.22 |
| ring finger and CCCH-type domains 2 | Rc3h2 | -4.22 |
| glycerol-3-phosphate acyltransferase, mitochondrial | Gpam | -4.21 |
| YTH domain family, member 3 | Ythdf3 | -4.21 |
| chromosome 5 open reading frame 51 | C5orf51 | -4.2 |
| lysine (K)-specific demethylase 7A | Kdm7a | -4.17 |
| WD repeat and FYVE domain containing 3 | Wdfy3 | -4.17 |
| strawberry notch homolog 1 | Sbno1 | -4.13 |
| ankyrin 2, neuronal | Ank2 | -4.11 |
| Midasin | Mdn1 | -4.06 |
| phosphoinositide kinase, FYVE finger containing | Pikfyve | -4.04 |
| decapping mRNA 2 | Dcp2 | -4.03 |
| neurexin-1-beta | [Nrxn1](http://www.ncbi.nlm.nih.gov/omim/600565) | -4.03 |
| phospholipase C, beta 1 (phosphoinositide-specific) | Plcb1 | -4.02 |
| opioid binding protein/cell adhesion molecule-like | Opcml | -3.98 |
| myotubularin related protein 9 | Mtmr9 | -3.97 |
| sema domain, immunoglobulin domain (Ig), short basic domain, secreted, (semaphorin) 3D | Sema3d | -3.96 |
| bromodomain and WD repeat domain containing 1 | Brwd1 | -3.94 |
| EPH receptor A3 | Epha3 | -3.93 |
| K(lysine) acetyltransferase 6A | Kat6a | -3.93 |
| SECIS binding protein 2-like | Secisbp2l | -3.93 |
| dicer 1, ribonuclease type III | Dicer1 | -3.91 |
| lysine (K)-specific demethylase 5A | Kdm5a | -3.91 |
| neurotrophic tyrosine kinase, receptor, type 2 | Ntrk2 | -3.9 |
| dipeptidyl-peptidase 8 | Dpp8 | -3.89 |
| nuclear receptor subfamily 1, group D, member 2 | Nr1d2 | -3.89 |
| ATPase, class V, type 10D | Atp10d | -3.88 |
| kelch domain containing 10 | Klhdc10 | -3.88 |
| glypican-5 isoform X4 | [Gpc5](http://www.ncbi.nlm.nih.gov/omim/602446) | -3.86 |
| solute carrier family 8 (sodium/calcium exchanger), member 1 | Slc8a1 | -3.86 |
| phosphatidylinositol-3,4,5-trisphosphate-dependent Rac exchange factor 2 | Prex2 | -3.85 |
| solute carrier family 12 (potassium/chloride transporter), member 6 | Slc12a6 | -3.85 |
| ubiquitin specific peptidase 32 | Usp32 | -3.85 |
| kinesin family member 3A | Kif3a | -3.84 |
| notch 2 | Notch2 | -3.84 |
| sel-1 suppressor of lin-12-like | Sel1l | -3.84 |
| teneurin transmembrane protein 3 | Tenm3 | -3.84 |
| FAT atypical cadherin 3 | Fat3 | -3.81 |
| integrin, alpha V | Itgav | -3.8 |
| phospholipase D1, phosphatidylcholine-specific | Pld1 | -3.79 |
| chromosome 17 open reading frame 85 | C17orf85 | -3.78 |
| calmodulin binding transcription activator 1 | Camta1 | -3.76 |
| unc-80 homolog | Unc80 | -3.76 |
| solute carrier family 1 (glial high affinity glutamate transporter), member 3 | Slc1a3 | -3.75 |
| sperm associated antigen 9 | Spag9 | -3.74 |
| membrane protein, palmitoylated 5 (MAGUK p55 subfamily member 5) | Mpp5 | -3.73 |
| zinc finger protein 106 | Znf106 | -3.73 |
| roundabout, axon guidance receptor, homolog 2 | Robo2 | -3.72 |
| mindbomb E3 ubiquitin protein ligase 1 | Mib1 | -3.71 |
| MYC binding protein 2, E3 ubiquitin protein ligase | Mycbp2 | -3.71 |
| furry | Fry | -3.7 |
| microtubule-actin crosslinking factor 1 | Macf1 | -3.69 |
| baculoviral IAP repeat containing 6 | Birc6 | -3.68 |
| fibronectin type III domain containing 3A | Fndc3a | -3.68 |
| ceramide synthase 6 | Cers6 | -3.67 |
| Rap guanine nucleotide exchange factor (GEF) 2 | Rapgef2 | -3.67 |
| senataxin | Setx | -3.67 |
| gap junction protein, alpha 1, 43kDa | Gja1 | -3.65 |
| centrosomal protein 170kDa | Cep170 | -3.64 |
| N(alpha)-acetyltransferase 25, NatB auxiliary subunit | Naa25 | -3.64 |
| teneurin transmembrane protein 2 | Tenm2 | -3.63 |
| AF4/FMR2 family, member 4 | Aff4 | -3.62 |
| cytochrome P450, family 4, subfamily V, polypeptide 2 | Cyp4v2 | -3.61 |
| LIM and calponin homology domains 1 | Limch1 | -3.61 |
| ELOVL fatty acid elongase 2 | Elovl2 | -3.6 |
| integrin, beta 8 | Itgb8 | -3.59 |
| RNA binding motif protein 27 | Rbm27 | -3.57 |
| myocyte enhancer factor 2C | Mef2c | -3.55 |
| ubiquitin specific peptidase 24 | Usp24 | -3.55 |
| mbt domain containing 1 | Mbtd1 | -3.54 |
| Ral GTPase activating protein, beta subunit (non-catalytic) | Ralgapb | -3.53 |
| phosphodiesterase 5A, cGMP-specific | Pde5a | -3.52 |
| kinesin family member 5C | Kif5c | -3.51 |
| nuclear receptor subfamily 3, group C, member 1 (glucocorticoid receptor) | Nr3c1 | -3.51 |
| dpy-19-like 4 | Dpy19l4 | -3.5 |
| SH3-domain GRB2-like (endophilin) interacting protein 1 | Sgip1 | -3.49 |
| ATPase, class V, type 10B | Atp10b | -3.48 |
| family with sequence similarity 73, member A | Fam73a | -3.48 |
| homeodomain interacting protein kinase 1 | Hipk1 | -3.48 |
| mannosyl (alpha-1,3-)-glycoprotein beta-1,4-N-acetylglucosaminyltransferase, isozyme A | Mgat4a | -3.48 |
| dedicator of cytokinesis 4 | Dock4 | -3.44 |
| gamma-aminobutyric acid (GABA) A receptor, beta 3 | Gabrb3 | -3.44 |
| neuron navigator 3 | Nav3 | -3.44 |
| zinc finger, MYM-type 4 | Zmym4 | -3.44 |
| ankyrin repeat and IBR domain containing 1 | Ankib1 | -3.42 |
| ubiquitin specific peptidase 34 | Usp34 | -3.42 |
| roundabout, axon guidance receptor, homolog 1 | Robo1 | -3.41 |
| family with sequence similarity 13, member B | Fam13b | -3.4 |
| cytoplasmic polyadenylation element binding protein 4 | Cpeb4 | -3.39 |
| mutated in colorectal cancers | Mcc | -3.38 |
| M-phase phosphoprotein 9 | Mphosph9 | -3.37 |
| Rho-related BTB domain containing 3 | Rhobtb3 | -3.37 |
| glutamate receptor, ionotropic, AMPA 3 | Gria3 | -3.36 |
| chromodomain helicase DNA binding protein 2 | Chd2 | -3.34 |
| inositol 1,4,5-trisphosphate receptor, type 1 | Itpr1 | -3.33 |
| neurofibromin 1 | Nf1 | -3.33 |
| transmembrane protein 47 | Tmem47 | -3.33 |
| AVL9 homolog | Avl9 | -3.29 |
| protein tyrosine phosphatase, non-receptor type 11 | Ptpn11 | -3.28 |
| dpy-19-like 3 | Dpy19l3 | -3.27 |
| intersectin 2 | Itsn2 | -3.27 |
| transcription factor 4 | Tcf4 | -3.27 |
| K(lysine) acetyltransferase 6B | Kat6b | -3.26 |
| glutamate receptor, ionotropic, AMPA 2 | Gria2 | -3.23 |
| neuronal cell adhesion molecule | Nrcam | -3.23 |
| tropomodulin 2 (neuronal) | Tmod2 | -3.23 |
| family with sequence similarity 13, member A | Fam13a | -3.22 |
| striatin interacting protein 2 | Strip2 | -3.21 |
| helicase with zinc finger | Helz | -3.2 |
| CDC42 binding protein kinase alpha (DMPK-like) | Cdc42bpa | -3.19 |
| glycerol-3-phosphate dehydrogenase 2 (mitochondrial) | Gpd2 | -3.19 |
| zinc finger CCCH-type containing 11A | Zc3h11a | -3.18 |
| protein phosphatase, Mg2+/Mn2+ dependent, 1K | Ppm1k | -3.17 |
| KDEL (Lys-Asp-Glu-Leu) containing 2 | Kdelc2 | -3.16 |
| transmembrane emp24 protein transport domain containing 5 | Tmed5 | -3.14 |
| UBX domain protein 2B | Ubxn2b | -3.14 |
| autophagy related 2B | Atg2b | -3.11 |
| biorientation of chromosomes in cell division 1-like 1 | Bod1l1 | -3.11 |
| QKI, KH domain containing, RNA binding | Qki | -3.11 |
| RAB GTPase-ACTIVATING PROTEIN 1 | [Rabgap1](http://www.ncbi.nlm.nih.gov/omim/615882) | -3.11 |
| ninein (GSK3B interacting protein) | Nin | -3.1 |
| Rho guanine nucleotide exchange factor (GEF) 12 | Arhgef12 | -3.09 |
| TRAF2 and NCK interacting kinase | Tnik | -3.09 |
| YTH domain containing 2 | Ythdc2 | -3.08 |
| ankyrin repeat domain 17 | Ankrd17 | -3.07 |
| transferrin receptor | Tfrc | -3.06 |
| ubiquitination factor E4A | Ube4a | -3.06 |
| ubiquitin specific peptidase 13 | Usp13 | -3.02 |
| glutamine--fructose-6-phosphate transaminase | Gfpt1 | -2.99 |
| dedicator of cytokinesis 9 | Dock9 | -2.87 |
