# Supplementary material for: Transcriptome Analysis Identifies Key Metabolic Changes in the Hooded Seal (Cystophora cristata) Brain in Response to Hypoxia and Reoxygenation
Source: PLoS One. 2017 Jan 3;12(1):e0169366. doi: 10.1371/journal.pone.0169366 (PMC5207758; doi:10.1371/journal.pone.0169366)
Supplement: S2 Table — Significantly up- (A) and downregulated (B) genes in seal brain slices after 1 h hypoxia followed by 20 min reoxygenation (N = 3 each). (DOC) [file pone.0169366.s010.doc]

**S3 Table.** Significantly up- (A) and downregulated (B) genes in seal brain slices after 1 h hypoxia followed by 20 min reoxygenation (N = 3 each).

| **A. Upregulated genes** | **Gene symbol** | **Fold change** |
| --- | --- | --- |
| Interleukin-1β | Il1b | 425.38 |
| FOS-like antigen 1 | Fosl1 | 374.32 |
| thyrotropin-releasing hormone | Trh | 111.86 |
| chemokine (C-C motif) ligand 3 | Ccl3 | 51.97 |
| v-maf avian musculoaponeurotic fibrosarcoma oncogene homolog F | Maff | 48.23 |
| chemokine (C-C motif) ligand 4 | Ccl4 | 44.6 |
| growth-regulated alpha protein-like | [Cxcl1](http://en.wikipedia.org/wiki/CXCL1) | 38.1 |
| BCL2-related protein A1 | Bcl2a1 | 35.03 |
| interleukin 1 receptor antagonist | Il1rn | 30.69 |
| activating transcription factor 3 | Atf3 | 28.73 |
| plasminogen activator, urokinase receptor | Plaur | 24.75 |
| Shisa family member 8 | Shisa8 | 13.53 |
| FBJ murine osteosarcoma viral oncogene homolog B | Fosb | 11.55 |
| adrenomedullin | Adm | 11.1 |
| zinc finger CCCH-type containing 12A | Zc3h12a | 10.56 |
| intercellular adhesion molecule 1 | Icam1 | 10.48 |
| solute carrier family 1 (neutral amino acid transporter), member 5 | Slc1a5 | 9.33 |
| growth arrest and DNA-damage-inducible, gamma | Gadd45g | 9.15 |
| chemokine (C-C motif) ligand 2 | Ccl2 | 8.93 |
| ZFP36 ring finger protein | Zfp36 | 7.75 |
| transgelin 2 | Tagln2 | 7.44 |
| AT rich interactive domain 5A (MRF1-like) | Arid5a | 7.41 |
| growth arrest and DNA-damage-inducible, beta | Gadd45b | 7.18 |
| protein phosphatase 1, regulatory subunit 15A | Ppp1r15a | 6.66 |
| predicted gene 11175 | Gm11175 | 5.9 |
| delta-like 4 | Dll4 | 5.73 |
| heme oxygenase (decycling) 1 | Hmox1 | 5.69 |
| protein QIL-1 | Qil1 | 5.32 |
| interferon regulatory factor 1 | Irf1 | 5.25 |
| lamin A/C | Lmna | 4.89 |
| BTG family, member 2 | Btg2 | 4.53 |
| nuclear receptor subfamily 4, group A, member 1 | Nr4a1 | 4.47 |
| B-cell CLL/lymphoma 3 | Bcl3 | 4.2 |
| Ets2 repressor factor | Erf | 3.75 |

| **B. Downregulated genes** | **Gene symbol** | **Fold change** |
| --- | --- | --- |
| kinesin family member 11 | Kif11 | -9.32 |
| zinc finger, DBF-type containing 2 | Zdbf2 | -8.95 |
| low density lipoprotein receptor-related protein 5 | [Lrp5](http://omim.org/entry/603506?search=low density lipoprotein receptor-related protein 5-like &highlight=like density receptorrelated 5like lipoprotein low proteinaceous protein) | -8.64 |
| centrosomal protein 85kDa-like | Cep85l | -8.16 |
| RAR-related orphan receptor A | Rora | -7.14 |
| formin 1 | Fmn1 | -6.95 |
| large tumor suppressor kinase 1 | Lats1 | -6.84 |
| zinc finger protein 197 | Znf197 | -6.66 |
| tet methylcytosine dioxygenase 2 | Tet2 | -6.60 |
| matrix metallopeptidase 16 (membrane-inserted) | Mmp16 | -5.93 |
| solute carrier family 4 (sodium bicarbonate cotransporter), member 4 | Slc4a4 | -5.73 |
| leucyl/cystinyl aminopeptidase | Lnpep | -5.71 |
| v-erb-b2 avian erythroblastic leukemia viral oncogene homolog 4 | Erbb4 | -5.70 |
| RPTOR independent companion of MTOR, complex 2 | Rictor | -5.66 |
| leukemia inhibitory factor receptor alpha | Lifr | -5.64 |
| replication timing regulatory factor 1 | Rif1 | -5.59 |
| transmembrane protein 168 | Tmem168 | -5.54 |
| Dmx-like 1 | Dmxl1 | -5.49 |
| leishmanolysin-like (metallopeptidase M8 family) | Lmln | -5.27 |
| ecotropic viral integration site 5 protein homolog | Evi5 | -5.19 |
| ATP-binding cassette, sub-family A (ABC1), member 6 | Abca6 | -5.18 |
| ATPase type 13A4 | Atp13a4 | -5.15 |
| potassium voltage-gated channel, subfamily H (eag-related), member 7 | Kcnh7 | -5.15 |
| pleckstrin homology domain interacting protein | Phip | -5.13 |
| ubiquitin specific peptidase 37 | Usp37 | -5.13 |
| aquaporin 4 | Aqp4 | -5.12 |
| cell division cycle 7 | Cdc7 | -5.09 |
| bromodomain and WD repeat domain containing 1 | Brwd1 | -5.05 |
| G protein-coupled receptor 98 | Gpr98 | -5.00 |
| chromodomain helicase DNA binding protein 9 | Chd9 | -4.99 |
| UDP-glucose glycoprotein glucosyltransferase 1 | Uggt1 | -4.88 |
| syntaxin binding protein 5-like | Stxbp5l | -4.85 |
| lysine (K)-specific demethylase 7A | Kdm7a | -4.78 |
| clock circadian regulator | Clock | -4.77 |
| SMG1 homolog, phosphatidylinositol 3-kinase-related kinase (C. elegans) | Smg1 | -4.76 |
| family with sequence similarity 199, X-linked | Fam199x | -4.75 |
| polyhomeotic homolog 3 (Drosophila) | Phc3 | -4.75 |
| paternally expressed 3 | Peg3 | -4.74 |
| PTC7 protein phosphatase homolog (S. cerevisiae) | Pptc7 | -4.74 |
| ER degradation enhancer, mannosidase alpha-like 3 | Edem3 | -4.73 |
| ceramide synthase 6 | Cers6 | -4.63 |
| family with sequence similarity 126, member A | Fam126a | -4.55 |
| natural killer cell triggering receptor | Nktr | -4.53 |
| RAR-related orphan receptor B | Rorb | -4.52 |
| TROVE domain family, member 2 | Trove2 | -4.51 |
| family with sequence similarity 171, member B | Fam171b | -4.48 |
| TAO kinase 1 | Taok1 | -4.48 |
| lysosomal trafficking regulator | Lyst | -4.47 |
| solute carrier family 4, sodium bicarbonate cotransporter, member 7 | Slc4a7 | -4.43 |
| ring finger and CCCH-type domains 2 | Rc3h2 | -4.42 |
| integrin, alpha V | Itgav | -4.41 |
| YTH domain family, member 3 | Ythdf3 | -4.36 |
| sodium channel, voltage-gated, type I, alpha subunit | Scn1a | -4.35 |
| sodium channel, voltage-gated, type II, alpha subunit | Scn2a | -4.33 |
| ATPase, Cu++ transporting, alpha polypeptide | Atp7a | -4.29 |
| dpy-19-like 4 (C. elegans) | Dpy19l4 | -4.28 |
| thyroid hormone receptor interactor 11 | Trip11 | -4.28 |
| protein tyrosine phosphatase, receptor type, D | Ptprd | -4.27 |
| RNA binding motif protein 27 | Rbm27 | -4.24 |
| family with sequence similarity 178, member A | Fam178a | -4.23 |
| mediator complex subunit 13 | Med13 | -4.23 |
| metaxin 3 | Mtx3 | -4.19 |
| remodeling and spacing factor 1 | Rsf1 | -4.18 |
| thrombospondin, type I, domain containing 7A | Thsd7a | -4.15 |
| integrin, beta 8 | Itgb8 | -4.14 |
| solute carrier family 1 (glial high affinity glutamate transporter), member 2 | Slc1a2 | -4.13 |
| vacuolar protein sorting 13 homolog C (S. cerevisiae) | Vps13c | -4.12 |
| ATPase, aminophospholipid transporter (APLT), class I, type 8A, member 1 | Atp8a1 | -4.11 |
| AT rich interactive domain 4B (RBP1-like) | Arid4b | -4.10 |
| baculoviral IAP repeat containing 8 | Xiap | -4.08 |
| KIAA1033 | Kiaa1033 | -4.08 |
| zinc finger, matrin-type 3 | Zmat3 | -4.08 |
| SECIS binding protein 2-like | Secisbp2l | -4.07 |
| ArfGAP with coiled-coil, ankyrin repeat and PH domains 2 | Acap2 | -4.03 |
| bromodomain and WD repeat domain containing 3 | Brwd3 | -4.03 |
| glutamate receptor, ionotropic, delta 2 | Grid2 | -4.03 |
| dipeptidyl-peptidase 8 | Dpp8 | -4.01 |
| transient receptor potential cation channel, subfamily M, member 3 | Trpm3 | -4.01 |
| DENN/MADD domain containing 4C | Dennd4c | -3.99 |
| gap junction protein, alpha 1, 43kDa | Gja1 | -3.98 |
| phosphoinositide kinase, FYVE finger containing | Pikfyve | -3.96 |
| gamma-aminobutyric acid (GABA) A receptor, beta 1 | Gabrb1 | -3.93 |
| neurexin 1 | Nrxn1 | -3.93 |
| ATP-binding cassette, sub-family A (ABC1), member 1 | Abca1 | -3.92 |
| FRY-like | Fryl | -3.92 |
| Rap guanine nucleotide exchange factor (GEF) 6 | Rapgef6 | -3.91 |
| phospholipase D1, phosphatidylcholine-specific | Pld1 | -3.90 |
| lysine (K)-specific demethylase 5A | Kdm5a | -3.88 |
| phospholipase C, beta 1 (phosphoinositide-specific) | Plcb1 | -3.88 |
| RAB11 family interacting protein 2 (class I) | Rab11fip2 | -3.86 |
| interleukin 6 signal transducer | Il6st | -3.85 |
| DAZ interacting zinc finger protein 3 | Dzip3 | -3.83 |
| leucine-rich repeat containing G protein-coupled receptor 4 | Lgr4 | -3.83 |
| transportin 1 | Tnpo1 | -3.81 |
| centrosomal protein 170kDa | Cep170 | -3.80 |
| sorting nexin 13 | Snx13 | -3.77 |
| ATPase, class V, type 10B | Atp10b | -3.75 |
| ubiquitin protein ligase E3 component n-recognin 1 | Ubr1 | -3.75 |
| dpy-19-like 3 (C. elegans) | Dpy19l3 | -3.74 |
| strawberry notch homolog 1 (Drosophila) | Sbno1 | -3.74 |
| zinc finger with KRAB and SCAN domains 1 | Zkscan1 | -3.72 |
| exportin 4 | Xpo4 | -3.71 |
| phosphatidylinositol-4,5-bisphosphate 3-kinase, catalytic subunit alpha | Pik3ca | -3.71 |
| spectrin repeat containing, nuclear envelope 1 | Syne1 | -3.71 |
| diaphanous-related formin 2 | Diaph2 | -3.67 |
| mbt domain containing 1 | Mbtd1 | -3.64 |
| sperm associated antigen 9 | Spag9 | -3.63 |
| cytochrome P450, family 4, subfamily V, polypeptide 2 | Cyp4v2 | -3.62 |
| TAF2 RNA polymerase II, TATA box binding protein (TBP)-associated factor, 150kDa | Taf2 | -3.62 |
| ubiquitin protein ligase E3 component n-recognin 2 | Ubr2 | -3.62 |
| mannosidase, alpha, class 2A, member 1 | Man2a1 | -3.59 |
| MYC binding protein 2, E3 ubiquitin protein ligase | Mycbp2 | -3.59 |
| A kinase (PRKA) anchor protein 6 | Akap6 | -3.58 |
| nuclear receptor subfamily 1, group D, member 2 | Nr1d2 | -3.57 |
| phosphodiesterase 5A, cGMP-specific | Pde5a | -3.55 |
| ATPase, Ca++ transporting, plasma membrane 1 | Atp2b1 | -3.54 |
| kinesin family member 3A | Kif3a | -3.54 |
| atlastin GTPase 3 | Atl3 | -3.47 |
| nuclear receptor subfamily 3, group C, member 1 (glucocorticoid receptor) | Nr3c1 | -3.47 |
| RAB GTPase activating protein 1-like | Rabgap1l | -3.47 |
| solute carrier family 1 (glial high affinity glutamate transporter), member 3 | Slc1a3 | -3.46 |
| EPH receptor A3 | Epha3 | -3.45 |
| kinesin family member 21A | Kif21a | -3.45 |
| LIM and senescent cell antigen-like domains 1 | Lims1 | -3.43 |
| transferrin receptor | Tfrc | -3.43 |
| ATP-binding cassette, sub-family C (CFTR/MRP), member 13 | Abcc13 | -3.37 |
| ELOVL fatty acid elongase 5 | Elovl5 | -3.36 |
| plexin C1 | Plxnc1 | -3.36 |
| WD repeat and FYVE domain containing 3 | Wdfy3 | -3.36 |
| mesoderm induction early response 1, transcriptional regulator | Mier1 | -3.35 |
| myocyte enhancer factor 2C | Mef2c | -3.35 |
| contactin 1 | Cntn1 | -3.34 |
| solute carrier organic anion transporter family, member 1C1 | Slco1c1 | -3.34 |
| Rho-related BTB domain containing 3 | Rhobtb3 | -3.32 |
| cell division cycle 27 | Cdc27 | -3.29 |
| ubiquitin specific peptidase 34 | Usp34 | -3.29 |
| cytoplasmic linker associated protein 2 | Clasp2 | -3.27 |
| mannosidase, alpha, class 1A, member 2 | Man1a2 | -3.27 |
| itchy E3 ubiquitin protein ligase | Itch | -3.25 |
| baculoviral IAP repeat containing 6 | Birc6 | -3.24 |
| zinc finger protein 106 | Znf106 | -3.24 |
| QKI, KH domain containing, RNA binding | Qki | -3.22 |
| ELOVL fatty acid elongase 2 | Elovl2 | -3.21 |
| collagen, type IV, alpha 3 (Goodpasture antigen) binding protein | Col4a3bp | -3.20 |
| glutamate receptor, ionotropic, AMPA 2 | Gria2 | -3.20 |
| FCH domain only 2 | Fcho2 | -3.19 |
| inhibitor of Bruton agammaglobulinemia tyrosine kinase | Ibtk | -3.19 |
| LIM and calponin homology domains 1 | Limch1 | -3.19 |
| folliculin interacting protein 2 | Fnip2 | -3.16 |
| membrane protein, palmitoylated 5 (MAGUK p55 subfamily member 5) | Mpp5 | -3.16 |
| cell adhesion molecule L1-like | Chl1 | -3.15 |
| DCC netrin 1 receptor | Dcc | -3.14 |
| mediator complex subunit 23 | Med23 | -3.11 |
| SUN domain containing ossification factor | Suco | -3.11 |
| transcription factor 4 | Tcf4 | -3.09 |
| neurofibromin 1 | Nf1 | -3.07 |
| tropomodulin 2 (neuronal) | Tmod2 | -3.03 |
| Ral GTPase activating protein, alpha subunit 1 (catalytic) | Ralgapa1 | -3.01 |
| ninein (GSK3B interacting protein) | Nin | -3.00 |
| SUZ12 polycomb repressive complex 2 subunit | Suz12 | -2.97 |
