# Supplementary material for: Transcriptome Analysis Identifies Key Metabolic Changes in the Hooded Seal (Cystophora cristata) Brain in Response to Hypoxia and Reoxygenation
Source: PLoS One. 2017 Jan 3;12(1):e0169366. doi: 10.1371/journal.pone.0169366 (PMC5207758; doi:10.1371/journal.pone.0169366)
Supplement: S3 Table — The ENSEMBL identifiers of the ferret genome are given. (DOC) [file pone.0169366.s011.doc]

**S3 Table. List of enzymes and other genes involved in the energy metabolism.** The ENSEMBL identifiers of the ferret nuclear genome are given, if available. The mean RPKM values of the normoxia, hypoxia and hypoxia-reoxygenation brain slices of the hooded seal are given. Note that RPKM values of the nuclear and mitochondrially encoded genes are not directly comparable.

| **Metabolic Pathway** | **Enzymes/Transporters** | | **Gene symbol** | **Ensembl identifier (nuclear genes only)** | **Mean RPKM (normoxia)** | | **Mean RPKM (hypoxia)** | **Mean RPKM (reoxygenation)** |  |
| --- | --- | --- | --- | --- | --- | --- | --- | --- | --- |
| Glycolysis, gluconeogenesis, glycogen synthesis | Hexokinase isoenzymes: | |  |  |  |  | |  | |
|  |  | Hexokinase 1 | *Hk1* | ENSMPUG00000004457 | 76.72 | 56.43 | | 69.09 | |
|  |  | Hexokinase 2 | *Hk2* | ENSMPUG00000008342 | RPKM <1 |  | |  | |
|  |  | Hexokinase 3 | *Hk3* | ENSMPUG00000012331 | RPKM <1 |  | |  | |
| Glycolysis, gluconeogenesis | Glucose-6-phosphate isomerase | | *Gpi* | ENSMPUG00000007520 | 512.01 | 409.33 | | 486.78 | |
|  | Phosphofructokinase isoenzymes: | |  |  |  |  | |  | |
|  |  | Phosphofructokinase muscle-type | *Pfkm* | ENSMPUG00000000441 | 247.4 | 164.44 | | 205.93 | |
|  |  | Phosphofructokinase liver-type | *Pfkl* | ENSMPUG00000010091 | 25.19 | 43.63 | | 42.83 | |
|  |  | Phosphofructokinase platelet-type | *Pfkp* | ENSMPUG00000016228 | 77.46 | 65.27 | | 75.11 | |
| Glycolysis, gluconeogenesis | Aldolase isoenzymes: | |  |  |  |  | |  | |
|  |  | Aldolase A | *AldoA* | ENSMPUG00000012563 | 858.47 | 1304.66 | | 1364.74 | |
|  |  | Aldolase B | *AldoB* | ENSMPUG00000006916 | 2.47 | 1.58 | | 1.71 | |
|  |  | Aldolase C | *AldoC* | ENSMPUG00000014221 | 988.63 | 1140.49 | | 1172.09 | |
|  | Triose phosphate isomerase | | *Tpi1* | ENSMPUG00000016774 | 569.79 | 854.39 | | 902.2 | |
|  | Glyceraldehyde-3-phosphate dehydrogenase | | *Gapdh* | ENSMPUG00000016891 | 1204.92 | 1835.54 | | 1911.91 | |
|  | Phosphoglycerate kinase isoenzymes: | |  |  |  |  | |  | |
|  |  | Phosphoglycerate kinase 1 | *Pgk1* | ENSMPUG00000000085 | 367.52 | 291.03 | | 309.32 | |
|  |  | Phosphoglycerate kinase 2 | *Pgk2* | ENSMPUG00000009179 | RPKM <1 |  | |  | |
|  | Phosphoglycerate mutase isoenzymes: | |  |  |  |  | |  | |
|  |  | Phosphoglycerate mutase 1 (brain) | *Pgam1* | ENSMPUG00000017045 | 332.07 | 200.09 | | 252.48 | |
|  |  | Phosphoglycerate mutase 2 (muscle) | *Pgam2* | ENSMPUG00000018207 | 4.29 | 9.93 | | 11.21 | |
|  | Enolase isoenzymes: | |  |  |  |  | |  | |
|  |  | Enolase 1 (alpha) | *Eno1* | ENSMPUG00000006382 | 178.5 | 169.09 | | 187.57 | |
|  |  | Enolase 2, (gamma, neuronal) | *Eno2* | ENSMPUG00000016769 | 127.42 | 153.29 | | 167.04 | |
|  |  | Enolase 3 (beta, muscle) | *Eno3* | ENSMPUG00000010972 | 5.2 | 8.57 | | 8.45 | |
|  |  | Enolase 4 | *Eno4* | ENSMPUG00000003414 | 1.46 | 0.72 | | 1.18 | |
| Glycolysis | Pyruvate kinase isoenzymes: | |  |  |  |  | |  | |
|  |  | Pyruvate kinase, liver and red blood cells | *Pklr* | ENSMPUG00000005509 | RPKM <1 |  | |  | |
|  |  | Pyruvate kinase, muscle | *Pkm* | ENSMPUG00000017126 | 301.73 | 390.1 | | 398.92 | |
| Glycolysis, gluconeogenesis | Lactate dehydrogenase isoenzymes | |  |  |  |  | |  | |
|  |  | Lactate dehydrogenase A | *Ldha* | ENSMPUG00000013730 | 74.69 | 52.93 | | 61.37 | |
|  |  | Lactate dehydrogenase B | *Ldhb* | ENSMPUG00000000172 | 599.56 | 670.87 | | 607.68 | |
| Gluconeogenesis | Pyruvate carboxylase | | *Pc* | ENSMPUG00000003573 | 28.78 | 49.37 | | 45.16 | |
|  | Phosphoenolpyruvate carboxykinase isoenzymes: | |  |  |  |  | |  | |
|  |  | Phosphoenolpyruvate carboxykinase 1 (soluble) | *Pck1* | ENSMPUG00000006661 | RPKM <1 |  | |  | |
|  |  | Phosphoenolpyruvate carboxykinase 2 (mitochondrial) | *Pck2* | ENSMPUG00000006229 | 2.27 | 3.24 | | 3.5 | |
| Gluconeogenesis, glycogen metabolism | Glucose-6-phosphatase complex subunits: | |  |  |  |  | |  | |
|  |  | Glucose-6-phosphatase, catalytic subunit | *G6pc* | ENSMPUG00000009965 | RPKM <1 |  | |  | |
|  |  | Glucose-6-phosphatase catalytic subunit 2 | *G6pc2* | ENSMPUG00000000733 | RPKM <1 |  | |  | |
|  |  | Glucose 6 phosphatase catalytic subunit 3 | *G6pc3* | ENSMPUG00000009547 | 6.36 | 10.07 | | 9.5 | |
|  |  | Solute carrier family 37 a 4 | *Slc37a4* | ENSMPUG00000004984 | 2.79 | 4.15 | | 3.93 | |
| Gluconeogenesis | Fructose-1,6-bisphosphatase isoenzymes: | |  |  |  |  | |  | |
|  |  | Fructose-1,6-bisphosphatase 1 | *Fbp1* | ENSMPUG00000006664 | RPKM <1 |  | |  | |
|  |  | Fructose-1,6-bisphosphatase 2 | *Fbp2* | ENSMPUG00000006656 | RPKM <1 |  | |  | |
| Glycogen metabolism | Phosphoglucomutase isoenzymes | |  |  |  |  | |  | |
|  |  | Phosphoglucomutase 1 | *Pgm1* | ENSMPUG00000008105 | 56.39 | 42.61 | | 51.52 | |
|  |  | Phosphoglucomutase 2 | *Pgm2* | ENSMPUG00000005926 | RPKM <1 |  | |  | |
|  |  | Phosphoglucomutase 3 | *Pgm3* | ENSMPUG00000005460 | 28.82 | 15.19 | | 13.34 | |
|  |  | Phosphoglucomutase 5 | *Pgm5* | ENSMPUG00000009659 | 1.93 | 2.03 | | 1.9 | |
|  | UDP-glucose pyrophosphorylase 2 | | *Ugp2* | ENSMPUG00000005966 | 56.03 | 29.25 | | 27.42 | |
|  | UDP-glycogen synthase isoenzymes: | |  |  |  |  | |  | |
|  |  | UDP-glycogen synthase 1 (muscle) | *Gys1* | ENSMPUG00000003291 | 8.17 | 8.79 | | 8.88 | |
|  |  | UDP-glycogen synthase 2 (liver) | *Gys2* | ENSMPUG00000000192 | RPKM <1 |  | |  | |
|  | Glucan (1,4-alpha-) branching enzyme 1 | | *Gbe1* | ENSMPUG00000014957 | 4.29 | 2.42 | | 1.84 | |
|  | Glycogen phosphorylase isoenzymes: | |  |  |  |  | |  | |
|  |  | Glycogen phosphorylase brain-type | *Pygb* | ENSMPUG00000015641 | 131.12 | 136 | | 140.08 | |
|  |  | Glycogen phosphorylase muscle-type | *Pygm* | ENSMPUG00000012740 | 37.24 | 41.02 | | 34.46 | |
|  |  | Glycogen phosphorylase liver-type | *Pygl* | ENSMPUG00000005218 | 7.83 | 4.99 | | 5.33 | |
|  | Amylo-alpha-1, 6-glucosidase | | *Agl* | ENSMPUG00000012276 | 13.52 | 3.85 | | 3.38 | |
| Pyruvate decarboxylation | Pyruvate dehydrogenase complex subunits: | |  |  |  |  | |  | |
|  |  | Pyruvate dehydrogenase (lipoamide) alpha 1 | *Pdha1* | ENSMPUG00000003269 | 102.03 | 41.85 | | 51.89 | |
|  |  | Pyruvate dehydrogenase beta | *Pdhb* | ENSMPUG00000016896 | 83.23 | 78.39 | | 67.48 | |
|  |  | Pyruvate dehydrogenase | *Pdhx* | ENSMPUG00000011686 | 23.18 | 10.87 | | 11.8 | |
|  |  | Dihydrolipoamide S-acetyltransferase | *Dlat* | ENSMPUG00000003657 | 20.29 | 7.97 | | 10.77 | |
|  |  | Dihydrolipoamide dehydrogenase | *Dld* | ENSMPUG00000018242 | 85.61 | 50.22 | | 42.07 | |
| Tricarboxylic acid cycle | Citrate synthase | | *Cs* | ENSMPUG00000001323 | 98.27 | 58.42 | | 74.81 | |
|  | Aconitase 2, mitochondrial | | *Aco2* | ENSMPUG00000016396 | 228.96 | 249.97 | | 253.25 | |
|  | Isocitrate dehydrogenase isoenzymes: | |  |  |  |  | |  | |
|  |  | Isocitrate dehydrogenase 3 (NAD+) gamma | *Idh3g* | ENSMPUG00000008781 | 22.31 | 31.27 | | 32.96 | |
|  |  | Isocitrate dehydrogenase 3 (NAD+) beta | *Idh3b* | ENSMPUG00000009107 | 70.51 | 81.56 | | 87.02 | |
|  |  | Isocitrate dehydrogenase 3 (NAD+) alpha | *Idh3a* | ENSMPUG00000011142 | 8.65 | 6.97 | | 6.47 | |
|  | α-Ketoglutarate dehydrogenase complex subunits: | |  |  |  |  | |  | |
|  |  | Oxoglutarate dehydrogenase (lipoamide) | *Ogdh* | ENSMPUG00000018194 | 32.07 | 45.87 | | 46.54 | |
|  |  | Dihydrolipoamide S-succinyltransferase | *Dlst* | ENSMPUG00000009271 | 19.74 | 14.8 | | 16.86 | |
|  |  | Dihydrolipoamide dehydrogenase | *Dld* | ENSMPUG00000018242 | 85.61 | 50.22 | | 42.07 | |
| Tricarboxylic acid cycle | Succinyl coenzyme A ligase subunits: | |  |  |  |  | |  | |
|  |  | Succinate-CoA Ligase, α-Subunit | *Suclg1* | ENSMPUG00000008566 | 34.16 | 42.93 | | 37.96 | |
|  |  | Succinate-CoA Ligase, ADP-Forming, β-Subunit | *Sucla2* | ENSMPUG00000001488 | 49.76 | 30.81 | | 26.83 | |
|  |  | Succinate-CoA Ligase, GDP-Forming, β-Subunit | *Suclg2* | ENSMPUG00000016950 | 4.36 | 3.03 | | 2.66 | |
| Tricarboxylic acid cycle, electron transfer chain, oxidative phosphorylation | Succinate dehydrogenase (SDH) complex subunits/Mitochondrial respiratory complex II: | | | |  |  | |  | |
|  |  | SDH subunit A, flavoprotein (Fp) | *Sdha* | ENSMPUG00000010324 | 148.03 | 112.16 | | 129.32 | |
|  |  | SDH subunit B, iron sulfur (Ip) | *Sdhb* | ENSMPUG00000016248 | 79.1 | 115.67 | | 96.13 | |
|  |  | SDH subunit C, integral membrane protein, 15kDa | *Sdhc* | ENSMPUG00000014439 | 52.51 | 73.77 | | 64.69 | |
|  |  | SDH subunit D, integral membrane protein | *Sdhd* | not annotated |  |  | |  | |
| Tricarboxylic acid cycle | Fumarate hydratase | | *Fh* | ENSMPUG00000015697 | 17.78 | 12.66 | | 15.8 | |
|  | Malate dehydrogenase 2, NAD (mitochondrial) | | *Mdh2* | ENSMPUG00000016000 | 101.46 | 181.5 | | 167.42 | |
| Electron transport chain, oxidative phosphorylation | Mitochondrial respiratory complex I/NADH dehydrogenase (ubiquinone) subunits: | | | |  |  | |  | |
|  |  | NADH Dehydrogenase 1 α-subcomplex 1 | *Ndufa1* | not annotated |  |  | |  | |
|  |  | NADH dehydrogenase 1 α-subcomplex 2 | *Ndufa2* | not annotated |  |  | |  | |
|  |  | NADH dehydrogenase 1 α-subcomplex 3 | *Ndufa3* | ENSMPUG00000018804 | 30.72 | 82.4 | | 84.3 | |
|  |  | NADH dehydrogenase 1 α-subcomplex 4 | *Ndufa4* | ENSMPUG00000014152 | 56.81 | 117.23 | | 100.97 | |
|  |  | NADH dehydrogenase 1 α-subcomplex 5 | *Ndufa5* | ENSMPUG00000006811 | 164.76 | 149.75 | | 125.44 | |
|  |  | NADH dehydrogenase 1 α-subcomplex 6 | *Ndufa6* | ENSMPUG00000016333 | 19.72 | 33.57 | | 31.35 | |
|  |  | NADH dehydrogenase 1 α-subcomplex 7 | *Ndufa7* | ENSMPUG00000007302 | 105.01 | 218.11 | | 226.12 | |
|  |  | NADH dehydrogenase 1 α-subcomplex 8 | *Ndufa8* | ENSMPUG00000007290 | 56.27 | 70.32 | | 77.72 | |
|  |  | NADH dehydrogenase 1 α-subcomplex 9 | *Ndufa9* | ENSMPUG00000009164 | 79.7 | 72.82 | | 84.18 | |
|  |  | NADH dehydrogenase 1 α-subcomplex 10 | *Ndufa10* | ENSMPUG00000000128 | 94.05 | 90.89 | | 103.72 | |
|  |  | NADH dehydrogenase 1 α-subcomplex 11 | *Ndufa11* | ENSMPUG00000006200 | 175.11 | 587.49 | | 618.14 | |
|  |  | NADH dehydrogenase 1 α-subcomplex 12 | *Ndufa12* | ENSMPUG00000007354 | 39.68 | 55.63 | | 52.77 | |
|  |  | NADH dehydrogenase 1 α-subcomplex 13 | *Ndufa13* | not annotated |  |  | |  | |
|  |  | NADH dehydrogenase 1 α/β subcomplex 1 | *Ndufab1* | ENSMPUG00000012142 | RPKM <1 |  | |  | |
|  |  | NADH dehydrogenase 1 β-subcomplex 1 | *Ndufb1* | not annotated |  |  | |  | |
|  |  | NADH dehydrogenase 1 β-subcomplex 2 | *Ndufb2* | ENSMPUG00000003423 |  |  | |  | |
|  |  | NADH dehydrogenase 1 β-subcomplex 3 | *Ndufb3* | not annotated |  |  | |  | |
|  |  | NADH dehydrogenase 1 β-subcomplex 4 | *Ndufb4* | ENSMPUG00000008272 |  |  | |  | |
|  |  | NADH dehydrogenase 1 β-subcomplex 5 | *Ndufb5* | ENSMPUG00000017216 | 28.45 | 35.02 | | 27.89 | |
|  |  | NADH dehydrogenase 1 β-subcomplex 6 | *Ndufb6* | ENSMPUG00000004295 | 128.04 | 225.4 | | 181.06 | |
|  |  | NADH dehydrogenase 1 β-subcomplex 7 | *Ndufb7* | ENSMPUG00000002959 | 87.57 | 254.57 | | 269.73 | |
|  |  | NADH dehydrogenase 1 β-subcomplex 8 | *Ndufb8* | ENSMPUG00000016959 | 65.53 | 145.98 | | 139.38 | |
|  |  | NADH dehydrogenase 1 β-subcomplex 9 | *Ndufb9* | ENSMPUG00000001303 | 111.64 | 219.2 | | 213.44 | |
|  |  | NADH dehydrogenase 1 β-subcomplex 10 | *Ndufb10* | ENSMPUG00000015557 | 157.05 | 327.99 | | 310.08 | |
|  |  | NADH dehydrogenase 1 β-subcomplex 11 | *Ndufb11* | ENSMPUG00000013112 | 112.12 | 279.66 | | 254.23 | |
|  |  | NADH dehydrogenase 1 subcomplex unknown 1 | *Ndufc1* | ENSMPUG00000016130 | 6.33 | 11.96 | | 11.21 | |
|  |  | NADH dehydrogenase 1 subcomplex unknown 2 | *Ndufc2* | ENSMPUG00000007921 | 203.05 | 580.63 | | 429.63 | |
|  |  | NADH dehydrogenase Fe-S protein 1 (NADH-coenzyme Q reductase) | *Ndufs1* | ENSMPUG00000000442 | 61.6 | 26.95 | | 23.55 | |
|  |  | NADH dehydrogenase Fe-S protein 2 (NADH-coenzyme Q reductase) | *Ndufs2* | ENSMPUG00000014279 | 168.03 | 335.57 | | 338.51 | |
|  |  | NADH dehydrogenase Fe-S protein 3 (NADH-coenzyme Q reductase) | *Ndufs3* | ENSMPUG00000003880 | 117.5 | 163.18 | | 160.86 | |
|  |  | NADH dehydrogenase Fe-S protein 4 (NADH-coenzyme Q reductase) | *Ndufs4* | ENSMPUG00000014335 | 17.92 | 21.04 | | 20.19 | |
|  |  | NADH dehydrogenase Fe-S protein 5 (NADH-coenzyme Q reductase) | *Ndufs5* | not annotated |  |  | |  | |
|  |  | NADH dehydrogenase Fe-S protein 6 (NADH-coenzyme Q reductase) | *Ndufs6* | ENSMPUG00000018120 | RPKM <1 |  | |  | |
|  |  | NADH dehydrogenase Fe-S protein 7 (NADH-coenzyme Q reductase) | *Ndufs7* | ENSMPUG00000008260 | 13.93 | 47.72 | | 42.15 | |
|  |  | NADH dehydrogenase Fe-S protein 8 (NADH-coenzyme Q reductase) | *Ndufs8* | ENSMPUG00000004193 | 93.72 | 233.71 | | 243.8 | |
|  |  | NADH dehydrogenase flavoprotein 1 | *Ndufv1* | ENSMPUG00000004041 | 110.52 | 194.99 | | 195.78 | |
|  |  | NADH dehydrogenase flavoprotein 2 | *Ndufv2* | ENSMPUG00000004710 | 117.5 | 103.36 | | 94.84 | |
|  |  | NADH dehydrogenase flavoprotein 3 | *Ndufv3* | ENSMPUG00000009667 | RPKM <1 |  | |  | |
|  |  | Mitochondrially encoded NADH dehydrogenase 1 | *MtNd1* |  | 108153 | 90926.8 | | 91821.9 | |
|  |  | Mitochondrially encoded NADH dehydrogenase 2 | *MtNd2* |  | 84279 | 62401.2 | | 54804.9 | |
|  |  | Mitochondrially encoded NADH dehydrogenase 3 | *MtNd3* |  | 51384.8 | 62670 | | 90997.5 | |
|  |  | Mitochondrially encoded NADH dehydrogenase 4 | *MtNd4* |  | 67106.5 | 43721.6 | | 42497.3 | |
|  |  | Mitochondrially encoded NADH dehydrogenase 4L | *MtNd4L* |  | 13.35 | 1.38 | | 6.73 | |
|  |  | Mitochondrially encoded NADH dehydrogenase 5 | *MtNd5* |  | 25614.6 | 11279.6 | | 10988.8 | |
|  |  | Mitochondrially encoded NADH dehydrogenase 6 | *MtNd6* |  | 42187.6 | 17243.3 | | 17695.7 | |
|  | *Mitochondrial respiratory complex III/Ubiquinone-cytochrome c reductase complex subunits* | | |  |  |  | |  | |
|  |  | cytochrome c-1 | *Cyc1* | ENSMPUG00000007180 | 96.4 | 283.76 | | 255.24 | |
|  |  | ubiquinol-cytochrome c reductase subunit X | *Uqcr10* | ENSMPUG00000013247 | 123.03 | 246.2 | | 240.25 | |
|  |  | ubiquinol-cytochrome c reductase subunit XI | *Uqcr11* | not annotated |  |  | |  | |
|  |  | ubiquinol-cytochrome c reductase binding protein | *Uqcrb* | not annotated |  |  | |  | |
|  |  | ubiquinol-cytochrome c reductase core protein I | *Uqcrc1* | ENSMPUG00000014540 | 111.56 | 222.22 | | 208.91 | |
|  |  | ubiquinol-cytochrome c reductase core protein II | *Uqcrc2* | ENSMPUG00000016850 | 22.23 | 16.55 | | 16.92 | |
|  |  | ubiquinol-cytochrome c reductase, Rieske iron-sulfur polypeptide 1 | *Uqcfrs1* | not annotated |  |  | |  | |
|  |  | ubiquinol-cytochrome c reductase hinge protein | *Uqcrh* | ENSMPUG00000012823 | 698.69 | 1253.26 | | 1161.85 | |
|  |  | ubiquinol-cytochrome c reductase subunit VII | *Uqcrq* | ENSMPUG00000010443 | 14.77 | 47.36 | | 35.78 | |
|  |  | mitochondrially encoded cytochrome b | *MtCyb* |  | 63832.2 | 47167.4 | | 49604.2 | |
|  | Mitochondrial respiratory complex IV/Cytochrome c oxidase subunits: | |  |  |  |  | |  | |
|  |  | cytochrome c oxidase subunit IV isoform 1 | *Cox4i1* | not annotated |  |  | |  | |
|  |  | cytochrome c oxidase subunit IV isoform 2 | *Cox4i2* | ENSMPUG00000017903 | RPKM <1 |  | |  | |
|  |  | cytochrome c oxidase subunit Va | *Cox5a* | ENSMPUG00000017314 | 257.83 | 322.24 | | 311.85 | |
|  |  | cytochrome c oxidase subunit Vb | *Cox5b* | ENSMPUG00000011327 | 59.03 | 163.41 | | 144.25 | |
|  |  | cytochrome c oxidase subunit VIa polypeptide 1 | *Cox6a1* | not annotated |  |  | |  | |
|  |  | cytochrome c oxidase subunit VIa polypeptide 2 | *Cox6a2* | ENSMPUG00000011359 | RPKM <1 |  | |  | |
|  |  | cytochrome c oxidase subunit VIb polypeptide 1 (ubiquitous) | *Cox6b1* | ENSMPUG00000006258 | 420.37 | 1217.41 | | 1143.88 | |
|  |  | cytochrome c oxidase subunit VIb polypeptide 2 (testis) | *Cox6b2* | ENSMPUG00000007546 | RPKM <1 |  | |  | |
|  |  | cytochrome c oxidase subunit VIc | *Cox6c* | not annotated |  |  | |  | |
|  |  | cytochrome c oxidase subunit VIIa polypeptide 1 | *Cox7a1* | ENSMPUG00000005626 | 121.61 | 285.41 | | 280.52 | |
|  |  | cytochrome c oxidase subunit VIIa polypeptide 2 | *Cox7a2* | not annotated |  |  | |  | |
|  |  | cytochrome c oxidase subunit VIIb | *Cox7b* | not annotated |  |  | |  | |
|  |  | cytochrome c oxidase subunit VIIb2 | *Cox7b2* | ENSMPUG00000018810 | RPKM <1 |  | |  | |
|  |  | cytochrome c oxidase subunit VIIc | *Cox7c* | not annotated |  |  | |  | |
|  |  | cytochrome c oxidase subunit VIIIA (ubiquitous) | *Cox8a* | not annotated |  |  | |  | |
|  |  | cytochrome c oxidase subunit VIIIC | *Cox8c* | not annotated |  |  | |  | |
|  |  | mitochondrially encoded cytochrome c oxidase I | *MtCo1* |  | 221619 | 299481 | | 187143 | |
|  |  | mitochondrially encoded cytochrome c oxidase II | *MtCo2* |  | 55744.1 | 61028.2 | | 56571.8 | |
|  |  | mitochondrially encoded cytochrome c oxidase III | *MtCo3* |  | 117298 | 127054 | | 138444 | |
|  | Mitochondrial respiratory complex V/ATP synthase (F1-F0) complex subunits: | |  |  |  |  | |  | |
|  |  | mitochondrial F1 complex alpha subunit 1, cardiac muscle | *Atp5a1* | ENSMPUG00000000923 | 151.51 | 153.63 | | 139.57 | |
|  |  | mitochondrial F1 complex beta polypeptide | *Atp5b* | ENSMPUG00000001507 | 681.65 | 699.32 | | 782.54 | |
|  |  | H+ transporting, mitochondrial F1 complex, gamma polypeptide 1 | *Atp5c1* | ENSMPUG00000016387 | 191.04 | 174.41 | | 169.54 | |
|  |  | mitochondrial F1 complex delta subunit | *Atp5d* | ENSMPUG00000008580 | 166.46 | 545.7 | | 550.91 | |
|  |  | mitochondrial F1 complex epsilon subunit | *Atp5e* | not annotated |  |  | |  | |
|  |  | mitochondrial Fo complex subunit B1 | *Atp5f1* | ENSMPUG00000004772 | 201.43 | 226.03 | | 197.68 | |
|  |  | mitochondrial Fo complex subunit C1 (subunit 9) | *Atp5g1* | ENSMPUG00000015341 | 89.54 | 265.68 | | 261.53 | |
|  |  | mitochondrial Fo complex subunit C2 (subunit 9) | *Atp5g2* | not annotated |  |  | |  | |
|  |  | mitochondrial Fo complex subunit C3 (subunit 9) | *Atp5g3* | ENSMPUG00000013986 | 370.65 | 562.48 | | 515.24 | |
|  |  | mitochondrial Fo complex subunit D | *Atp5h* | ENSMPUG00000014048 | 163.57 | 260.93 | | 244.11 | |
|  |  | mitochondrial Fo complex subunit E | *Atp5i* | ENSMPUG00000011414 | 369.53 | 886.54 | | 919.5 | |
|  |  | mitochondrial Fo complex subunit F6 | *Atp5j* | ENSMPUG00000001001 | 84.09 | 142.86 | | 121.52 | |
|  |  | mitochondrial Fo complex subunit F2 | *Atp5j2* | not annotated |  |  | |  | |
|  |  | mitochondrial Fo complex subunit G | *Atp5l* | ENSMPUG00000004622 | 334.15 | 784 | | 711.82 | |
|  |  | mitochondrial Fo complex subunit G2 | *Atp5l2* | not annotated |  |  | |  | |
|  |  | mitochondrial F1 complex subunit O | *Atp5o* | ENSMPUG00000001319 | 105.4 | 170.02 | | 139.76 | |
|  |  | ATPase inhibitory factor 1 | *Atpif1* | ENSMPUG00000015536 | 91.55 | 215.92 | | 178.73 | |
|  |  | mitochondrially encoded ATP synthase 6 | *MtAtp6* |  | 108344 | 101932 | | 115424 | |
|  |  | mitochondrially encoded ATP synthase 8 | *MtAtp8* |  | 49717.3 | 44413.4 | | 46477.9 | |
| Glycerol-3-phosphate shuttle | Glycerol-3-phosphate shuttle | |  |  |  |  | |  | |
|  |  | glycerol-3-phosphate dehydrogenase 1 (soluble) | *Gpd1* | ENSMPUG00000014346 | 12.43 | 17.43 | | 14.69 | |
|  |  | glycerol-3-phosphate dehydrogenase 2 (mitochondrial) | *Gpd2* | ENSMPUG00000000440 | 14.98 | 3.97 | | 4.74 | |
| Monocarboxylic acids transport | Monocarboxylate transporter (MCT) isoforms | | | |  |  | |  | |
|  |  | MCT1 | *Mct1* | ENSMPUG00000005243 | 1.03 | 0.5 | | 0.36 | |
|  |  | Solute carrier family 16 A member 7 (MCT2) | *Slc16a7* | ENSMPUG00000002081 | 2.25 | 0.79 | | 0.39 | |
|  |  | Solute carrier family 16 A member 3 (MCT4) | *Slc16a3* | ENSMPUG00000011135 | 3.03 | 6.68 | | 6.2 | |
